# Desmin Modulates Muscle Cell Adhesion and Migration

**DOI:** 10.3389/fcell.2022.783724

**Published:** 2022-03-08

**Authors:** Coralie Hakibilen, Florence Delort, Marie-Thérèse Daher, Pierre Joanne, Eva Cabet, Olivier Cardoso, Fany Bourgois-Rocha, Cuixia Tian, Eloy Rivas, Marcos Madruga, Ana Ferreiro, Alain Lilienbaum, Patrick Vicart, Onnik Agbulut, Sylvie Hénon, Sabrina Batonnet-Pichon

**Affiliations:** ^1^ Université de Paris, BFA, UMR 8251, CNRS, Paris, France; ^2^ Sorbonne Université, Institut de Biologie Paris-Seine (IBPS), CNRS UMR 8256, INSERM ERL U1164, Biological Adaptation and Ageing, Paris, France; ^3^ Université de Paris, MSC, UMR 7067, CNRS, Paris, France; ^4^ Department of Neurology, Cincinnati Children’s Hospital Medical Center, College of Medicine, University of Cincinnati, Cincinnati, OH, United States; ^5^ Servicio de Anatomia Patologica, Hospital Universitario Virgen Del Rocio, Sevilla, Spain; ^6^ Unidad de Neurologia Pediatrica, Hospital Universitario Virgen Del Rocio, Sevilla, Spain; ^7^ APHP, Centre de Référence Maladies Neuromusculaires Nord/Est/Ile-de-France, Groupe Hospitalier Pitié-Salpêtrière, Paris, France

**Keywords:** desmin, vinculin, focal adhesion, migration, myopathies, intermediate filaments

## Abstract

Cellular adhesion and migration are key functions that are disrupted in numerous diseases. We report that desmin, a type-III muscle-specific intermediate filament, is a novel cell adhesion regulator. Expression of p.R406W mutant desmin, identified in patients with desmin-related myopathy, modified focal adhesion area and expression of adhesion-signaling genes in myogenic C2C12 cells. Satellite cells extracted from desmin-knock-out (DesKO) and desmin-knock-in-p.R405W (DesKI-R405W) mice were less adhesive and migrated faster than those from wild-type mice. Moreover, we observed mislocalized and aggregated vinculin, a key component of cell adhesion, in DesKO and DesKI-R405W muscles. Vinculin expression was also increased in desmin-related myopathy patient muscles. Together, our results establish a novel role for desmin in cell-matrix adhesion, an essential process for strength transmission, satellite cell migration and muscle regeneration. Our study links the patho-physiological mechanisms of desminopathies to adhesion/migration defects, and may lead to new cellular targets for novel therapeutic approaches.

## Introduction

The cytoskeleton orchestrates many essential cell processes such as cellular division, mechanosensing, adhesion and motility. These functions derive from the three main structural networks: microfilaments (actin filaments), microtubules and intermediate filaments (IFs). Most studies focus on the role of microfilament or microtubule networks in cell adhesion and migration. However, new insights indicate an important role of the IF family in various cell types such as myoblasts (Etienne-Manneville, 2018; Agnetti et al., 2021; Kural Mangit et al., 2021).

The cell-specific IF network is often pictured as an integrator of microfilaments and microtubules via a complex set of cross-bridging proteins (Seifert et al., 1992). In all tissues, IFs form transcellular networks in direct interaction with specific cell–cell or cell–matrix junctions called desmosomes and hemidesmosomes (Troyanovsky et al., 1993; Mogensen et al., 1998). IFs contribute mainly to cell shape maintenance and cellular organelle anchoring and are involved in cell strength, adhesion and cohesion. IF are also important in cell division (Duarte et al., 2019) and resistance to stress (Etienne-Manneville, 2018). IFs seem to mediate key cell functions (i.e., cell adhesion and migration, organelle shaping and positioning) by providing cellular signposts through post-translational modifications (Omary et al., 2006; Pallari and Eriksson, 2007; Hyder et al., 2008).

Unlike microfilaments and microtubules, IFs are not polarized. IFs share a basic structure with a common tripartite organization characterized by a central alpha-helical coiled-coil-forming domain and non-alpha-helical ‘‘head’’ and ‘‘tail’’ domains of variable lengths and sequences (Strelkov et al., 2003). IFs self-assemble into filaments of ∼10-nm diameter (Herrmann et al., 1996). Yet, IF proteins exhibit tissue-specific expression: vimentin in cells derived from mesenchymal cells as fibroblasts or endothelial cells, desmin in muscle and cytokeratin generally in epithelial cells (Toivola et al., 2005). Keratin IFs have an important role in adhesion and cell–cell interactions through their association with hemidesmosomes and desmosomes (Osmani and Labouesse, 2015; Seltmann et al., 2015).

*In vitro,* cell adhesion and, in part, cell migration are controlled by integrins and focal adhesion (FA) formation. Among the 24 pairs of integrins described in vertebrates (Luo et al., 2007), α5β1 and α7β1 are the main pairs expressed in skeletal muscle cells. α5β1 and α7β1, respectively, function in adhesion to fibronectin and laminin, two essential components of the muscle extracellular matrix. FAs also involve vinculin, a 120-kDa cytoskeletal protein and major component of cell–cell and cell–extracellular matrix adhesion junctions (Bays and DeMali, 2017). In mature FAs, vinculin is crucial in the “molecular clutch” that mediates transmission of force from cytoplasmic F-actin to membrane-bound integrins. In adult striated muscle, FA complexes (called costameres) mediate connections between fibers and extracellular matrix (Pardo et al., 1983), thereby providing a substrate for assembly and maintenance of the muscle during mechanical constraints (contraction/relaxation) (Sparrow and Schöck, 2009; García-Pelagio et al., 2011). Costameres formed *in vitro* involve integrins and vinculin, but muscle cells also express an isoform of vinculin containing an additional 68-amino-acid insert within the tail domain, called metavinculin (Bays and DeMali, 2017). Expression of metavinculin depends on the differentiation state and increases with myogenesis (Saga et al., 1985).

Integrins are also increasingly linked to IFs as well. For instance, keratins could stabilize hemidesmosomes by regulating integrin turnover in keratinocytes (Seltmann et al., 2015). Similarly, in fibroblasts, vimentin, a type III IF that shares high homology with desmin, plays key roles in adhesion and migration by regulating integrin functions (Ivaska et al., 2007; Kim et al., 2016). The coalescence of vimentin IFs at mature FAs is required for endoplasmic spreading (Lynch et al., 2013). Finally synemin, a desmin-associated type VI IF, can interact with vinculin, metavinculin, talin and zyxin, thereby linking the heteropolymeric IFs to adhesion-type junctions within striated muscle cells, suggesting that it plays an important role in cytoskeleton component assembly and morphogenesis (Paulin et al., 2020; Russell, 2020).

The muscle-specific IF is desmin. During skeletal muscle differentiation, desmin progressively replaces vimentin and is commonly used as a differentiation marker. Importantly, to date more than 70 mutations in the desmin gene have been causally linked to rare neuromuscular diseases called desminopathies, associated either with abnormal desmin expression linked to muscle protein aggregation (Goldfarb et al., 1998; Dagvadorj et al., 2004; Fidziańska et al., 2005; Mavroidis et al., 2008; Pica et al., 2008; Fichna et al., 2014; Clemen et al., 2015), or with a total lack of desmin [4 families described, (Carmignac et al., 2009; Henderson et al., 2013)]. In both cases, muscles are disorganized, which triggers at least late skeletal muscle weakness. A well-established mouse model of desmin disruption (DesKO) demonstrated the importance of desmin in maintaining muscle integrity and function. DesKO mice are viable and fertile, but develop progressive myopathy associated with regeneration defects (Agbulut et al., 2001), loss of costamere anchorage and muscle fatigability (Li et al., 1996; Li et al., 1997; Joanne et al., 2021). We generated a new mouse knock-in model of a specific desmin missense mutation, p.R405W, corresponding to p.R406W in humans, which partially mimics the patient phenotype with desmin aggregates present in myofibrils (Batonnet-Pichon et al., 2017; Herrmann et al., 2020). Currently, there is no established link between desmin and skeletal muscle cell adhesion.

Using these two mouse models, we focused here on the potential link between desmin and cell adhesion and migration in undifferentiated muscle cells as well as in mature muscle. To do so, we developed stable cell lines under the desmin promoter to determine the impact of the p.R406W mutation on genome-wide expression. We then followed adhesion in C2C12 myoblasts overexpressing p.R405W mutant desmin, using vinculin as a well-established marker of adhesion. We then extracted satellite cells from KO or R405W KI mouse models and investigated the functional adhesion of shear-stressed satellite cells and their non-directed migration properties. Finally, we examined the expression level and localization of vinculin in muscle from patients with desminopathies and adult controls. Our findings reveal novel roles for desmin in muscle cell adhesion and migration, with important implications for clinical pathology.

## Materials and Methods

### Mouse Models

Generation and characterization of desmin knock-out mice (DesKO) were previously described (Li et al., 1996). DesKO mouse genotype was confirmed by polymerase chain reaction using primers 5′ TTG​GGG​TCG​CTG​CGG​TCT​AGC​C 3′, 5′ GGT​CGT​CTA​TCA​GGT​TGT​CAC​G 3′ and 5′ GAT​CGA​TCT​CGC​CAT​ACA​GCG​C 3′. For DesKI-R405W mice, genotypes were determined with primers 5′ CTG​GAG​GAG​GAG​ATC​CGA​CA 3′ and 5′ GGC​CCT​CGT​TAA​TTT​TCT​GC 3′.

All animal experiments were conducted with respect to animal health and well-being, and all procedures were approved by our institutional ethics committee (authorization CEB-16-2016).

### Cell Lines, Culture and Electroporation

C2C12 cells (ATCC) were grown in DMEM (Dulbecco’s Modified Eagle’s Medium, Life Technologies) supplemented with 10% fetal bovine serum (FBS, PAA Laboratories) and 1% penicillin/streptomycin (Life Technologies). pEGFP constructs (Charrier et al., 2016; Charrier et al., 2018) were electroporated into C2C12 cells using a Gene Pulser II (BioRad, Hercules, CA). Briefly, cells were trypsinized (Trypsin-EDTA, Life Technologies) for 5 min at 37°C and resuspended in complete DMEM medium at 2 × 10^6^ cells/ml. Then, 400 µl of the cell suspension were introduced in a 0.4-cm gap Gene Pulser Cuvette (BioRad) and submitted to 250 V, 1 mF for 25 ms. After electroporation, cells were plated on fibronectin-coated glass coverslips for 24 h before TIRFM microscopy.

For stable expression, we first replaced the pcDNA3 CMV promoter with the murine 4 kb desmin promoter, which contains all essential sites to induce expression in skeletal and cardiac muscle cells. Then 5′-Myc-tagged complete human *desmin* wild-type cDNA was subcloned. This plasmid also contains the puromycin resistance gene for selection. Mutated plasmid carrying the R406W mutation was then obtained using the Quick Change-XL site-directed mutagenesis kit (Stratagene), following the manufacturer’s instructions. All plasmids were sequenced (Eurofins, MWG). Stable C2C12 cells expressing WT or R406W mutant desmin were generated after nucleofection of these plasmids with Amaxa nucleofector (Lonza, Basel, Switzerland) according to the manufacturer’s instructions. Subsequently, puromycin (Euromedex, 1 μg/ml) selection was applied for 1 week. Proliferation selections yielded around thirty clones for each construct. Clones were chosen based on Myc-desmin expression as well as their proliferation and differentiation abilities.

Satellite cells were extracted from two gasctrocnemius and two plantaris muscles of 1-month-old DesKI-R405W (Batonnet-Pichon et al., 2017; Herrmann et al., 2020) and DesKO mice (Li et al., 1996). Our protocol was adapted from previous studies (Ohanna et al., 2005). Briefly, muscles were incubated 4 × 10 min in DMEM/F12 + Glutamax solution (Life Technologies) containing 1.5 mg/ml protease XIV (Sigma-Aldrich, Saint-Louis) and 1/500 primocin (InvivoGen) at 37°C under regular agitation. Following each incubation, tubes were centrifuged at 400 g for 30 s at room temperature. The first floating cells were eliminated, and the three other fractions containing satellite cells were diluted in 20% FBS medium and sieved with a 40-µm filter. Filtrate containing satellite cells was centrifuged for 5 min at 1,400 g at room temperature. Cells were then incubated in DMEM/F12 + Glutamax containing 20% FBS, 2% Ultroser (Pall Life Science, Portsmouth, United Kingdom), 8.6 ng/ml FGF2 (Life Technologies), 1/100 N2 (Life Technologies) and 1/500 primocin and seeded onto 6-well plates coated with 1/20 Matrigel (Corning, NY, United States) at 100 cells/cm^2^. After activation and proliferation, satellite cells were maintained in T150 flasks at 1,000 cells/cm^2^ on plastic covered with 1/20 Matrigel. For analysis, cells were seeded at the adapted concentration on fibronectin or laminin.

### Western Blotting

A total of 30 µg of muscle or cell extracts were loaded per well on a 10% acrylamide gel for SDS-PAGE. After migration, proteins were transferred onto nitrocellulose membranes (0.45 µm, Macherey Nagel), which were saturated 1 h with 5% non-fat milk in 0.5% Tween/PBS solution. Membranes were incubated with primary antibodies 1 h at room temperature or overnight at 4°C. Monoclonal mouse anti-vinculin (1/15,000, #V9264, Sigma-Aldrich), polyclonal rabbit anti-desmin (1/1,000, #C3956, Sigma-Aldrich), monoclonal rabbit anti-vimentin (1/2,500, #ab92547, abcam) and polyclonal rabbit anti-GAPDH (1/2,500, #G9545, Sigma-Aldrich) were used.

Isotype-specific anti-mouse or anti-rabbit secondary antibodies coupled with horseradish peroxidase (1/10,000, #31430 or #31460, Pierce, Thermo Scientific) were detected following incubation with Clarity Western ECL (BioRad) and visualized with a CCD camera (FUJI Las 4000 or Ai600, GE Healthcare).

### Gene Microarray Analyses

Total RNAs from the C2C12 skeletal myoblasts were purified using standard RNA extraction protocols (NucleoSpin RNA II, Macherey-Nagel). RNA concentration and integrity were determined using a Bioanalyzer 2100 (Agilent Technologies). Cy3-or Cy5-labeled cRNA was made using the Agilent Low RNA Input Linear Amp Kit (Agilent Technologies) following the manufacturer’s instructions, which employs a linear amplification method with T7 polymerase. Yields of cRNA and the dye-incorporation rate were measured with an ND-1000 Spectrophotometer (NanoDrop Technologies). Non-transfected control C2C12 cRNAs were labeled with Cy3 and experimental samples expressing wild-type or R406W mutant desmin C2C12 cRNAs were labeled with Cy5. The paired labeled Cy3/Cy5 cRNAs (825 ng) were mixed and hybridized overnight (17 h, 65°C) to Agilent Whole Mouse Genome Oligo Microarrays 4 × 44 K (Agilent Technologies). Fluorescence signals of the hybridized Agilent Oligo Microarrays were detected using Agilent’s DNA microarray scanner (Agilent Technologies) and analyzed using Agilent Feature Extraction Software and the Rosetta Resolver gene expression data analysis system (Rosetta Biosoftware). To highlight the most relevant pathways and genes, bioinformatics analyses were achieved. Gene ontology analysis and functional annotation of differentially expressed genes were performed using the DAVID web tool (https://david.ncifcrf.gov/summary.jsp). Gene annotations of Biological Process, Cellular Component and Molecular Function, together with annotations from the KEGG pathway database, were used, and only gene ontology terms that presented at least five genes in common with our gene expression matrix were considered in the analysis. Each *p*-value was directly calculated by the software.

### Cell Immunofluorescence

Cells were fixed with 2% paraformaldehyde (Santa Cruz Biotechnologies, Dallas, TX, United States) for 15 min at room temperature, then permeabilized with 0.5% Triton X-100 (Sigma-Aldrich) for 10 min and blocked with 4% BSA (Sigma-Aldrich). They were incubated with mouse monoclonal anti-vinculin primary antibody (1/150, Sigma-Aldrich) or mouse monoclonal anti-desmin antibody (1/100, DAKO, D33) for 1 h at room temperature. After three washes with PBS, cells were incubated 45 min with isotype-specific anti-mouse secondary antibody labeled with Alexa-488 (Molecular Probes) or anti-rabbit secondary antibody labeled with Alexa-568 (Molecular Probes). DNA was stained with Hoechst 33258 (1 μg/ml, Sigma-Aldrich) during secondary antibody incubation. Finally, cells were washed in PBS and mounted in Fluoromount medium (Interchim). Images were acquired by confocal microscopy (LSM700 Zeiss) at the imaging facility of the Functional and Adaptive Biology (BFA) unit.

### TIRFM

Electroporated cells were plated on fibronectin-coated glass coverslips 24 h before TIRF microscopy (Ti-Eclipse, NIKON). Cells were fixed and vinculin was immunostained as described above with anti-vinculin (1/150, Sigma-Aldrich) and then secondary antibody Alexa-568. The TIRFM system and image acquisition are controlled by MetaMorph software (Version 7.6, Molecular Device). The 25 × 25-mm glass coverslips were installed in a microscopy chamber and cells were covered with 50 µl of 1× PBS. The parameters of the TIRFM angle were previously determined on C2C12 cells. Cells of interest (carrying the desmin:GFP fusion) were identified by epifluorescence at 488-nm. Area and adhesion patches were analyzed using a macro developed by O. Cardoso (MSC Paris University, Supplementary Figure S1). Briefly, quantification was based on pixel binarization from a thresholding function in the ImageJ software. Area and patch number were then determined by the particle analysis function of the software.

### Adhesion Under Shear Stress

Satellite cells were plated 24 h before the experiment in a fibronectin-coated channel of an Ibitreat micro-slide VI 0.4 (Ibidi). DMEM/F12 + Glutamax (Life Technologies) supplemented with 4 mM EDTA (Sigma-Aldrich) was injected with a pump syringe (Standard Infusion Only PHD ULTRA™ Syringe Pumps, Harvard Apparatus, France) in the chamber slide at 218 ml/h as previously described (Modulevsky et al., 2012). Images of adhesive cells were taken every 15 s for 24 min with an Olympus IX83 microscope. Cells at each time point were counted with Fiji Cell Counter software with at least 80 cells per condition.

### Cellular Migration

Satellite cells were plated on fibronectin or laminin-coated 96-well plates 24 h before the experiment and medium was renewed 2 h before the start. Cells were placed at 37°C under 5% CO_2_ in a microscope enclosure. Bright field images were taken every 7 min for each position. Cell migration was followed with the Fiji Manual Tracking plugin. Cell persistence quantifies the ability of a cell to maintain its direction of motion. It is the ratio between its distance to origin at the end of the trajectory over the total distance travelled. Mean speed and persistence for each cell were calculated with Excel software. For each condition, at least 250 cells were counted.

### Preparation of Quadriceps and Soleus Muscles

Quadriceps and/or soleus muscles were removed from mice at 1, 3, 5 and 12 months of age after euthanizing by cervical dislocation. For immunohistochemical analysis, muscles were embedded in tragacanth gum (Fisher Scientific) and frozen by plunging in isopentane precooled with liquid nitrogen for at least 1 min. For western blotting, muscles were directly frozen in liquid nitrogen in microtubes, pulverized in a cryogenic mortar (Dominique Dutscher) and resuspended in RIPA buffer [50 mM Tris, 150 mM NaCl, 1% NP40, 5 mM EDTA, 1 mM NA_3_VO_4_, 10 mM NaF, 1 mM PMSF and anti-protease (Sigma-Aldrich)].

### Muscle Sections and Immunofluorescence Staining

Serial sections of 7-µm thickness were sliced using a CM1950 cryostat (Leica), recovered on Superfrost Plus microscope slides (Thermo Scientific) and stored at −80°C. Muscle sections were kept at room temperature for 15 min before staining. Endogenous fluorescence was prevented by treatment with 50 mM NH_4_Cl for 30 min. Sections were permeabilized in 0.5% Triton X100/PBS for 10 min, blocked in 4% bovine serum albumin (BSA, Sigma-Aldrich)/PBS and incubated with mouse monoclonal anti-vinculin (Sigma-Aldrich) or rabbit polyclonal anti-laminin (1/100, #L9393, Sigma-Aldrich) primary antibodies for 1 h at room temperature. Primary antibodies were detected by incubating sections with suitable secondary antibodies for 45 min. DNA was stained with 1 μg/ml Hoechst 33258 (Sigma-Aldrich) during secondary antibody incubation. Muscles were washed in PBS and mounted in Fluoromount medium (Interchim, San Diego, CA, United States). All images were captured using a digital camera mounted to a confocal laser scanning microscope (LSM700 ZEISS) at the imaging facility of the BFA unit.

### Patients and Human Biopsies

All patients presented a homozygous mutation in the *desmin* gene. The four patients were from Spain, United States (USA) and France. The control was from France. There were two biopsies from the same Spanish patients at 1 and 3 years old. The patient from the USA was a young girl deceased at around age of 20 (article in preparation). The French patients were previously presented (Carmignac et al., 2009). Muscle samples from all family members were obtained after informed consent for medical publications and presentations, in agreement with local ethics committees.

All human biopsies were frozen in nitrogen liquid and conserved at −80°C. Frozen biopsy samples were lysed following the same protocol as for mouse muscle: the lysis step was followed by a 2-h incubation with rotation at 4°C.

### Statistical Analysis

For microarray analysis, to identify differentially expressed genes with a fold change ≥2, one-way ANOVA was performed using a threshold *p*-value ≤ 0.01. For other statistical analyses, non-parametric statistical tests were carried out using GraphPadPrism software or R free software. A non-parametric ad hoc “nparcomp” test was performed for FA area comparison. Histograms were generated using GraphPadPrism software and a Mann-Whitney test was used for immunodetection results. A Kruskall-Wallis test followed by a Dunnet multiple comparisons post-test was used for the migration experiment. A Friedman test followed by a Dunnet multiple comparisons post-test was applied to the percentages of detachment from the shear stress experiment. Differences were considered significant at *p* < 0.05. All error bars correspond to the standard deviation (SD).

## Results

### R406W Mutant Desmin Overexpression Induces Modifications of Adhesion Pathways

**FIGURE 1:**
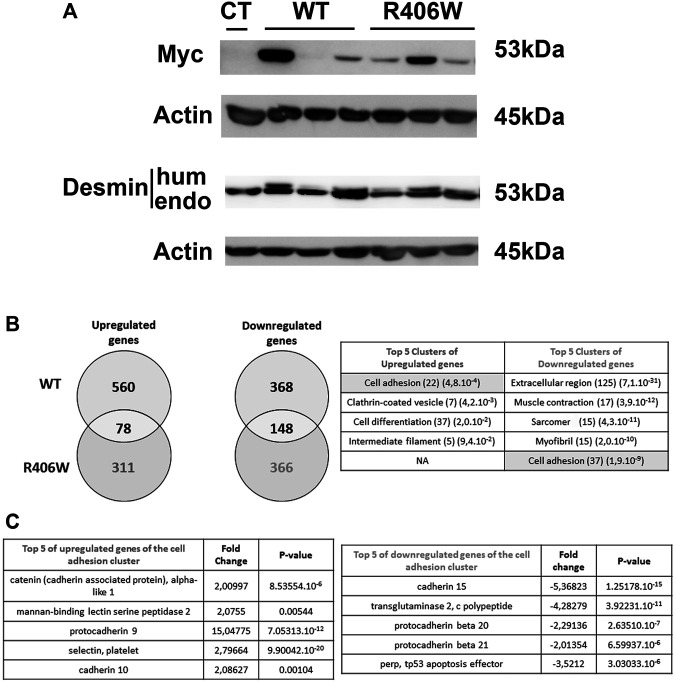
Human desmin expression and DNA chip analysis in stable cell lines. **(A)** The amounts of endogenous and human (Myc tagged) desmin in WT and p.R406W expressing clones were compared by Western blot. Actin was used as a loading control. **(B)** Genes that were upregulated or downregulated in C2C12 cells expressing R406W desmin or WT desmin were compared (right) and clustered using gene ontology enrichment analysis (left). The number of hits and *p*-values are respectively shown in brackets **(C)** Examples of up-regulated (right) or down-regulated (left) genes from the clusters determined in **(B)**.

To investigate the impact of desmin mutations on genome-wide expression, we first generated murine C2C12 myoblast cell lines stably expressing human wild-type or R406W desmin under the 4 Kb human desmin promoter. To distinguish between exogenous and endogenous desmin, a c-Myc tag was introduced at the 5′ end of the desmin cDNA. We selected three clones for each construct (WT or p.R406W) and monitored desmin expression by western blot. Myc-desmin was detected both in wild-type and R406W desmin expressing cells, but presented various expression levels, probably due to the location of the inserted transgene (Figure 1A). Interestingly, two bands corresponding to endogenous (mouse, lower band) and exogenous (human, higher band) desmin were revealed with anti-desmin specific antibodies, allowing us to quantify the ratio between the two forms. As previously reported (Delort et al., 2019), endogenous desmin was always more highly expressed than exogenous desmin (Figure 1A, the ratio of exogenous/endogenous varies from 0.1 to 0.7), corresponding to moderate desmin overexpression close to the pathophysiological conditions observed in patients.

We next performed transcriptomic profiling of C2C12 cells stably expressing wild-type and R406W desmin (using each clone expressing the same level of Myc-desmin WT or R406W with ratio exogenous/endogenous desmin around 0.5) in comparison to non-transfected C2C12 cells with Agilent Whole Mouse Genome Oligo Microarrays. In cells expressing wild-type desmin, 638 genes were up- and 516 genes were down-regulated compared to non-transfected cells, whereas in p.R406W-expressing cells, 389 genes were up and 514 genes were downregulated (Figure 1B, right). To identify specifically up and downregulated genes in mutant-expressing cells, we removed all mutually modified genes in our analysis. After this step, we specifically identified 311 genes that were up and 366 genes that were downregulated in p.R406W-expressing cells (Figure 1B, right).

The most relevant pathways and genes were identified by bioinformatic analyses. The top Gene Ontology categories included muscle contraction, myofibril, cell differentiation and interestingly, cell adhesion. The biological process of cell adhesion was found in both up-regulated and down-regulated clusters, indicating a strong perturbation of this function specifically in C2C12 cells expressing R406W desmin. Among these differentially expressed genes, we identified 37 down (*p* = 1.9e-9) and 22 upregulated (*p* = 4.8e-4) genes related to cell adhesion (see Figure 1B, left; Supplementary Table S1 for complete list), including genes of IF family (Supplementary Table S1, S2) and genes involved in cadherin or catenin related pathways (Figure 1C).

### Overexpression of Desmin Modulates Focal Adhesion Area in Electroporated C2C12 Cells

**FIGURE 2:**
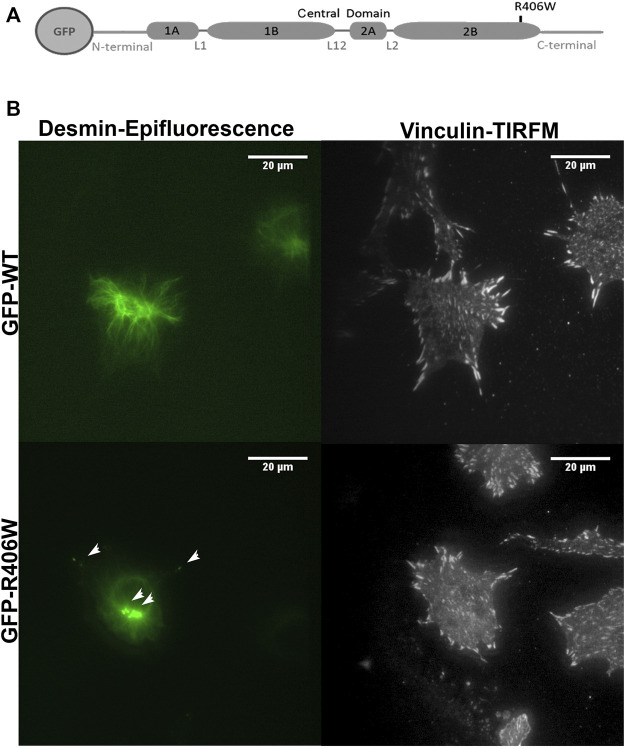
Desmin and vinculin morphology in C2C12 cells. **(A)** Representation of WT or R406W mutant desmin fused to GFP at the N-terminus. **(B)** GFP-WT desmin or GFP-R406W desmin were electroporated into C2C12 myoblasts. Vinculin was stained in red with Alexa-568. Epifluorescence images were taken 24 h after transfection. The p.R406W expressing cells show intracytoplasmic aggregates of desmin, unlike those expressing wild-type desmin. The cells were observed using a ×60 immersion objective. The scale bar corresponds to 20 μm.

To further explore the impact of desmin in the adhesion process, we focused on FAs. We electroporated murine C2C12 undifferentiated myoblasts carrying N-ter-GFP fused human WT desmin or R406W mutant desmin cDNA (Figure 2A). We first checked that the two constructs have similar expression levels (Supplementary Figure S2) and then analyzed the organization of the desmin network using epifluorescence and TIRFM (Figure 2B, the two analyses were performed on the same cells). Cells electroporated with wild-type desmin cDNA exhibit a well-organized desmin network, whereas cells electroporated with R406W desmin cDNA displayed aggregates near the nucleus and in the cytoplasm as expected (Figure 2B, arrowheads).

**FIGURE 3:**
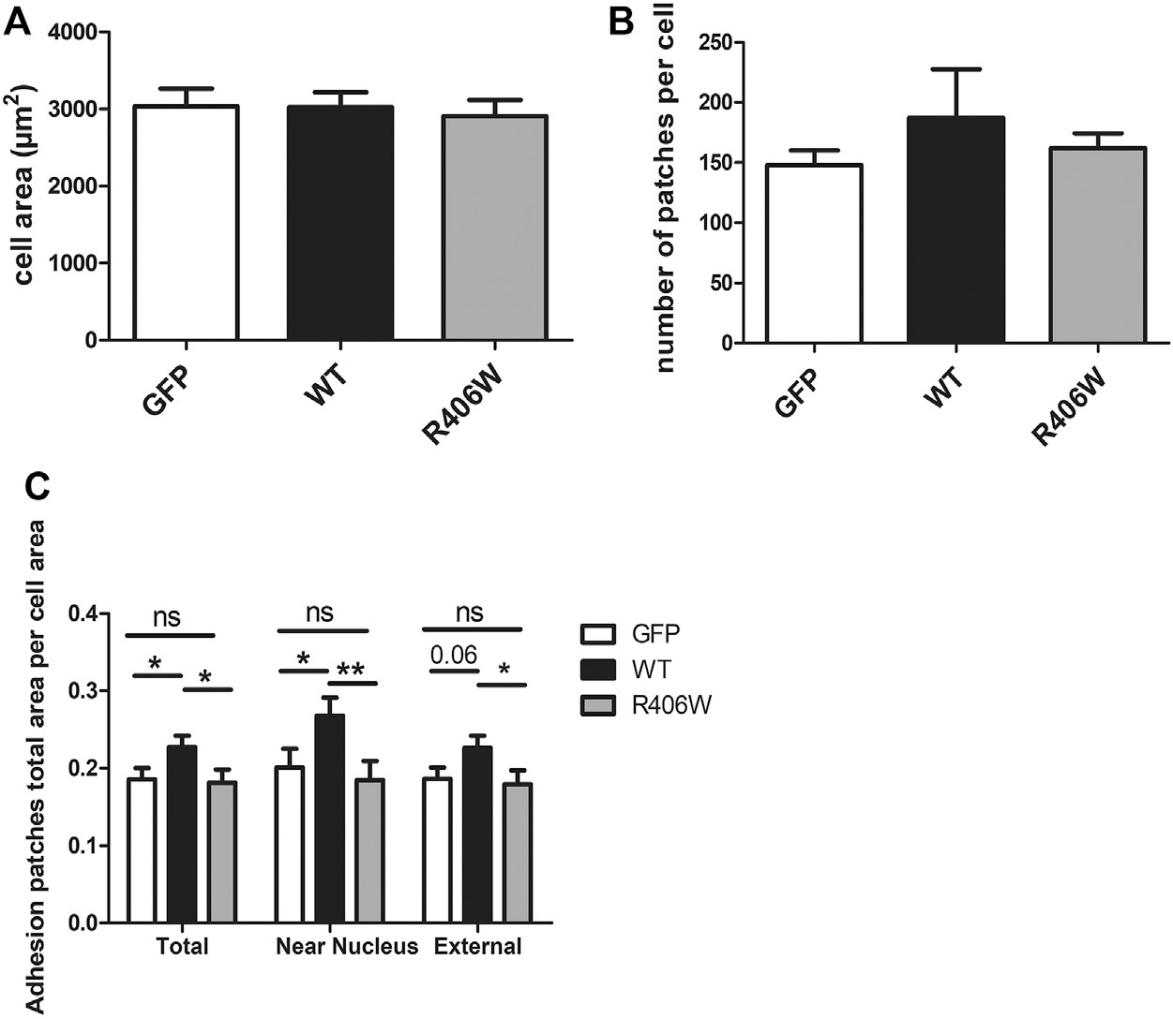
Effect of desmin mutation on the total area of focal adhesions in C2C12 cells. **(A)** Average area of cells electroporated with GFP, GFP-WT desmin or GFP-R406W desmin. **(B)** Average vinculin patch number per cell counted for the three cell lines and **(C)** Average total vinculin patch area per cell area for the three cell lines, for the entire cells (“Total”), for the region of the cells located under the nucleus (“Near nucleus”) and for the cell minus this nuclear zone (“External”). Kruskal-Wallis tests were performed. *p < 0.05, **p < 0.01. The measurements were performed with a home-designed ImageJ macro described in the *Materials and Methods* section and Supplementary Figure S1. These results represent the average of n > 40 cells per condition from three independent experiments.

To determine the impact of WT or mutated desmin overexpression on cell adhesion, we stained vinculin, an established marker of FA complexes (Figure 2B). As the number and area of vinculin patches reflect the formation and regulation of FAs, we first analyzed these parameters. In addition, we measured the total cell surface. Cells electroporated with GFP alone were used as a control (Figure 3).

None of the overexpressed forms of desmin resulted in a change in total cell area (Figure 3A) or in the number of FA patches per cell (Figure 3B). However, the total area of the patches, normalized by the cell area, was 22% larger for cells overexpressing WT desmin compared to that of cells expressing GFP alone (Figure 3C). Moreover, this effect was more pronounced in the central region of the cells, near the nucleus (33% increase), and less in the periphery (21% increase), where the effect of GFP-desWT expression was just beyond significance. On the contrary, such an increase was not observed with the expression of desR406W, suggesting a loss of function regarding FA modulation. The same results were obtained when staining paxillin instead of vinculin (data not shown).

### DesKO and DesKI Satellite Cells Exhibit Decreased Adhesive Properties on Fibronectin Substrate Under Hydrodynamic Flow

**FIGURE 4:**
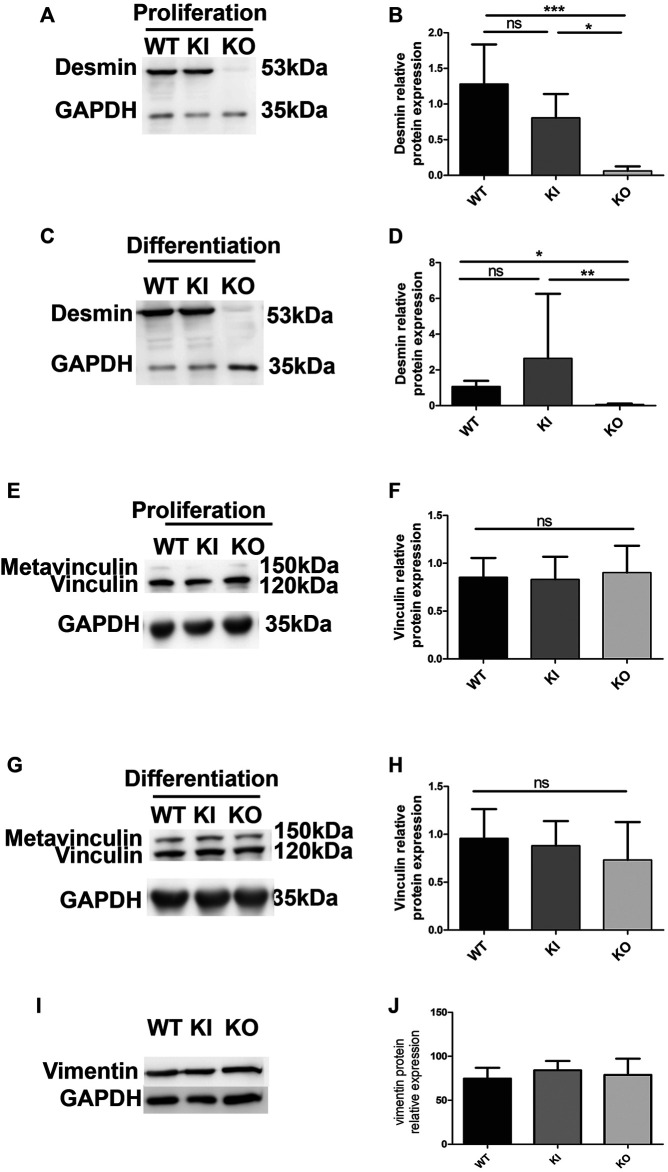
Relative protein expression of desmin, vinculin and vimentin in proliferating and differentiating satellite cells. Relative protein expression of desmin in proliferating **(A,B)** and differentiating **(C,D)** satellite cells was quantified by immunodetection and normalized by GAPDH protein expression. Each histogram represents the average of two lines per condition (3 for DesKI) and three independent experiments. The relative protein expression of vinculin was measured in proliferating **(E,F)** and differentiating **(G,H)** satellite cells by Western blot and normalized to the expression of GAPDH. The relative protein expression of vimentin was measured in proliferating **(I,J)** satellite cells by Western blot and normalized to the expression of GAPDH. Non-parametric Kruskal-Wallis tests were performed on the samples with: n.s, not significant; *p < 0.05; **p < 0.01 and ***p < 0.001.

To avoid overexpression of desmin, and to be closer to physiological conditions, we used satellite cells extracted from a knock-out mouse model of desmin and a homozygous knock-in mouse model carrying the p.R405W mutation (murine homologue of the human p.R406W mutation), respectively named DesKO (Li et al., 1996) and DesKI-R405W (Batonnet-Pichon et al., 2017; Herrmann et al., 2020). We first confirmed the absence of desmin expression in DesKO satellite cells (Figures 4A,B). Second, we followed desmin levels in proliferating (Figures 4A,B) and differentiating (Figures 4C,D) DesKI-R405W cells. As expected, desmin expression increases during differentiation. However, contrary to muscle (Herrmann et al., 2020), satellite cells had a similar amount of mutated or WT desmin, even during differentiation (Figures 4A–D).

Since modifications of the FA areas were observed in C2C12 myoblasts overexpressing desmin, we checked whether vinculin expression could be altered in satellite cells. Vinculin expression remained unchanged in all cell types and conditions (Figures 4E–H), as well as vimentin (Figures 4I,J), which is co-expressed with desmin in prolifating muscle cell.

**FIGURE 5:**
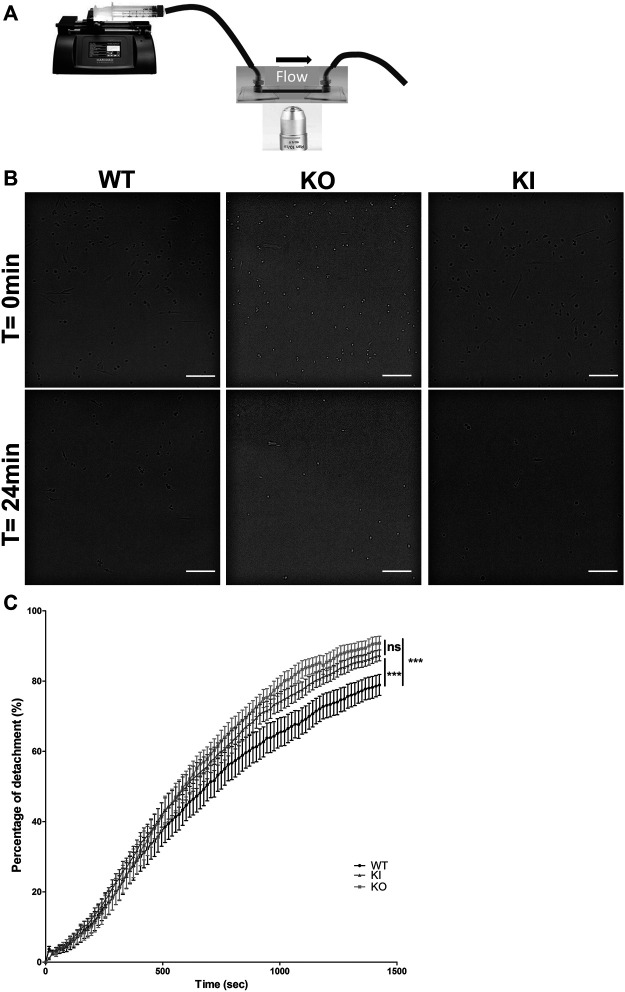
Detachment of satellite cells during the application of a shear flow. **(A)** A syringe-pump delivered a constant flow of medium through tubing on cells seeded on Ibidi slides. Images were acquired every 15 s during the experiment. **(B)** Satellite cells detached over time. After 24 min, the remaining cells were counted. More WT satellite cells remained adhered to the substrate than DesKO and DesKI-R405W satellite cells. Images were taken on an inverted microscope using a ×10 objective. Scale bar = 200 µm. **(C)** The number of cells remaining adhered to the substrate were counted to measure the percentage of detachment over time. A significant difference in detachment was measured between the WT satellite cells and the DesKO and DesKI-R405W mutant satellite cells. A non-parametric Friedman test was used: ***p < 0.001. The error bars correspond to the SD. The experiment was repeated 3 times with two independent satellites cells lines extracted from two mice each for WT and KO genotype and three independent satellites cells lines extracted from three mice for KI genotype.

To determine whether desmin could have a role in cellular adhesion, we performed functional analysis under shear stress in DesKO or DesKI-R405W contexts. Satellite cells were seeded on fibronectin-coated Ibitreat microslides and a constant flow was applied with a home-made setup (Figure 5A). After 24 min of shear stress, satellite cells were almost completely peeled off (Figure 5B). However, more WT cells seemed to be left attached than DesKI-R405W or DesKO cells. To confirm this qualitative observation, we determined the detachment rate by counting cells manually on each image (Figure 5C). From 0 to 500 s, all three cell types presented similar behaviors. In contrast, from 500 s, DesKO and DesKI-R405W satellite cells peeled off more significantly than the WT cells. Indeed, from 1,000 s, homozygous mutant cells (DesKO and DesKI-R405W) had a detachment rate 10% higher than that of WT cells. Finally, at the end of the experiment (24 min), 78.9% of the WT cells were detached from the substrate (Figure 5C) compared to 90.8% and 87.3% respectively of DesKO and DesKI-R405W cells. Altogether, this suggests that homozygous DesKO and DesKI-R405W satellite cells are less adhesive than WT cells on fibronectin.

### DesKO and DesKI-R405W Satellite Cells Exhibit Higher Migration Speed

**FIGURE 6:**
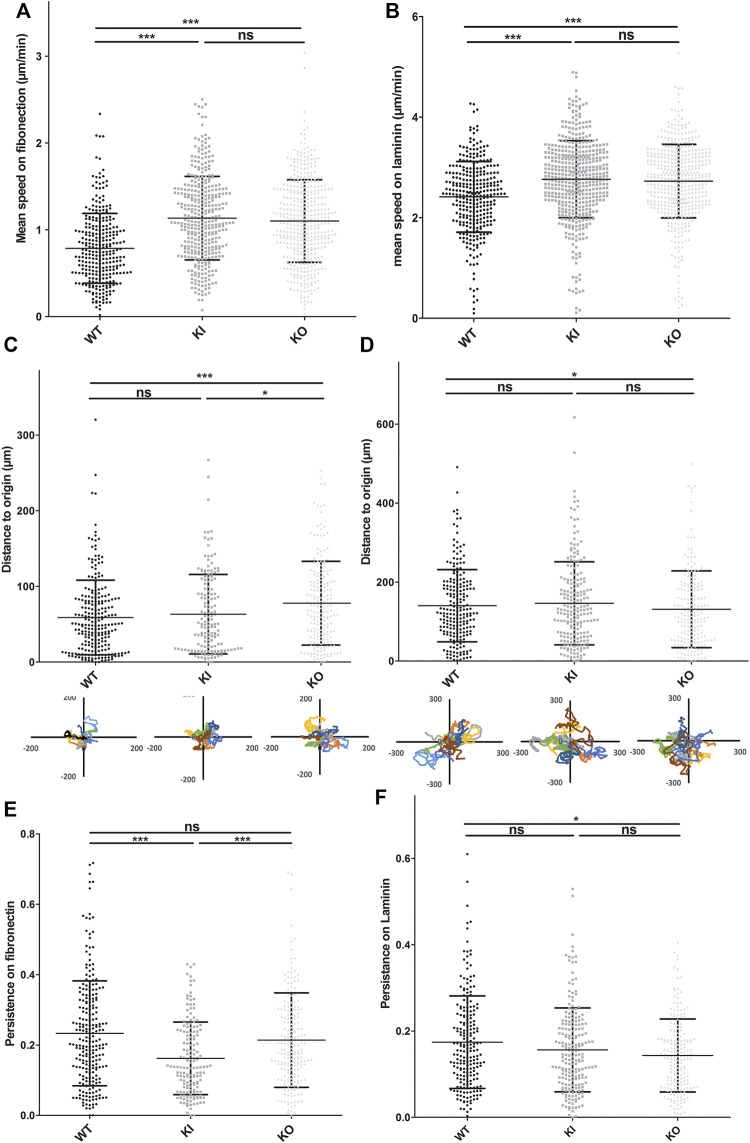
DesKO and DesKI-R405W cells move faster on fibronectin and laminin substrates. Cells were seeded on culture wells coated with fibronectin **(A,C,E)** or laminin **(B,D,F)**. Pictures were taken every 7 min. Cells were tracked by hand using ImageJ software and its manual tracking plugin. Each average represents two different cell extractions for each mice genotype, and experiments were repeated 3 times. with *n* > 150 cells and the error bars represent the SD. **(A,B)** The mean speed of each cell line is represented. The distance to the origin on fibronectin **(C)** and laminin **(D)** was calculated from the coordinates obtained during the migration follow-up with ImageJ software. WT DesKO and DesKI-R405W cell migration way was calculated with Excel software from a representative sample of eight cells per genotype. Cell persistence on fibronectin **(E)** and laminin **(F)** was calculated by their distance from the origin and their total migration distance. A Kruskal-Wallis test was performed, and the error bars correspond to the SD: n.s, not significant; *p < 0.05; ***p < 0.001

Satellite cells can migrate into mature muscle in regenerative conditions and in culture on different substrates. Migration and adhesion are related and interdependent processes. Thus, to determine whether desmin mutation can also affect cell migration, we used time-lapse to follow the non-directed motion of satellite cells on the two main substrates expressed in muscle: fibronectin and laminin. First we confirmed, as described *in vitro* (Siegel et al., 2009), that satellite cells present a higher migration rate on laminin than on fibronectin-coated surfaces (Supplementary Figure S3). Second, we compared WT to homozygous DesKO and DesKI-R405W cells on each type of substrate. On fibronectin, the average velocities of homozygous DesKO (1.1 μm min^−1^) and DesKI (1.13 μm min^−1^) cells were significantly higher (around 58%) than those of WT cells (0.79 μm min^−1^) (Figure 6A). No significant difference was observed between the two mutant cells.

Satellite cells migrated faster on laminin, with mean migration rates of 2.41, 2.73, and 2.76 μm min^−1^, respectively for WT, DesKO and DesKI-R405W cells (Figure 6B, Supplementary Videos S1/WT, S2/KI and S3/KO). Thus, DesKO and DesKI-R405W mutant satellite cells migrate about 15% faster than WT cells on laminin.

Together, these results suggest that desmin is involved in migration, and desmin modifications (loss or non-sense mutation) alter cell migratory properties.

The ability of cells to migrate in a directional way can be represented by cell persistence (see *Materials and Methods*). On fibronectin, the average persistence of WT, DesKO and DesKI-R405W cells was 0.23, 0.21 and 0.16, respectively (Figure 6E). Thus, DesKI-R405W cells had a 24% decrease in persistence compared to WT cells, suggesting less directionality. However, although DesKO cells migrated faster than WT cells on fibronectin, their directionalities were similar (Figure 6C).

On laminin, WT, DesKO, and DesKI-R405W cells had similar persistence (0.17, 0.14 and 0.16, respectively, less than 12% difference, Figure 6F).

### Vinculin Expression and Localization Are Altered in DesKO and DesKI-R405W Muscle

Finally, to confirm a link between desmin and markers of adhesion, we analyzed vinculin expression in mature muscle. In mouse models of desminopathies, as in patients, not all muscles are affected in the same way. In mice, the soleus, a slow posture muscle, is one of the muscles presenting the most pathological defects. Thus, we performed expression analysis on the soleus from DesKO and DesKI-R405W mice at ages presenting a well-established phenotype, with either a strong decrease in weight (DesKO, DesKI-R405W) or around the time of death for DesKI-R405W mice (Batonnet-Pichon et al., 2017; Herrmann et al., 2020).

**FIGURE 7:**
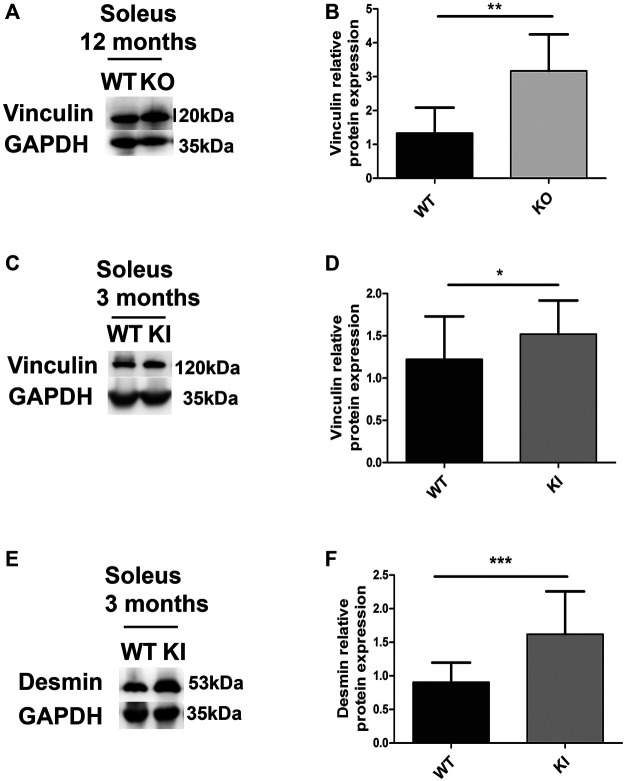
Relative vinculin and desmin protein expression in DesKO and DesKI-R405W mouse soleus. The amount of vinculin was measured in the soleus of mutant DesKO **(A,B)** and DesKI-R405W mice **(C,D)**. The amount of desmin was measured in the soleus of mutant DesKI-R405W mice **(E,F)**. Each histogram represents the average relative protein expression of four independent GAPDH-standardized Western blot experiments for 7 WT mice and 7 DesKO mice or 6 WT and 5 DesKI-R405W mice. A Mann-Whitney non-parametric test was performed, with *p < 0.05; **p < 0.01; ***p < 0.001. Error bars represent the SD.

The soleus of one-year-old DesKO mice had a 2.5-fold increase in vinculin expression compared to WT mice (Figures 7A,B). The same augmentation was also observed in the quadriceps of DesKO mice, in an age-dependent manner, without any changes in metavinculin quantity (Supplementary Figure S4). In the same way, the vinculin level was also increased in the soleus from 3-month-old DesKI-R405W mice compared to WT mice (Figures 7C,D). Furthermore, this increase was associated with a rise in the desmin level at this age (Figures 7E,F). Similar alterations of vinculin and desmin expression were found in the quadriceps muscles of 3-month-old DesKI-R405W mice (Supplementary Figure S5).

**FIGURE 8:**
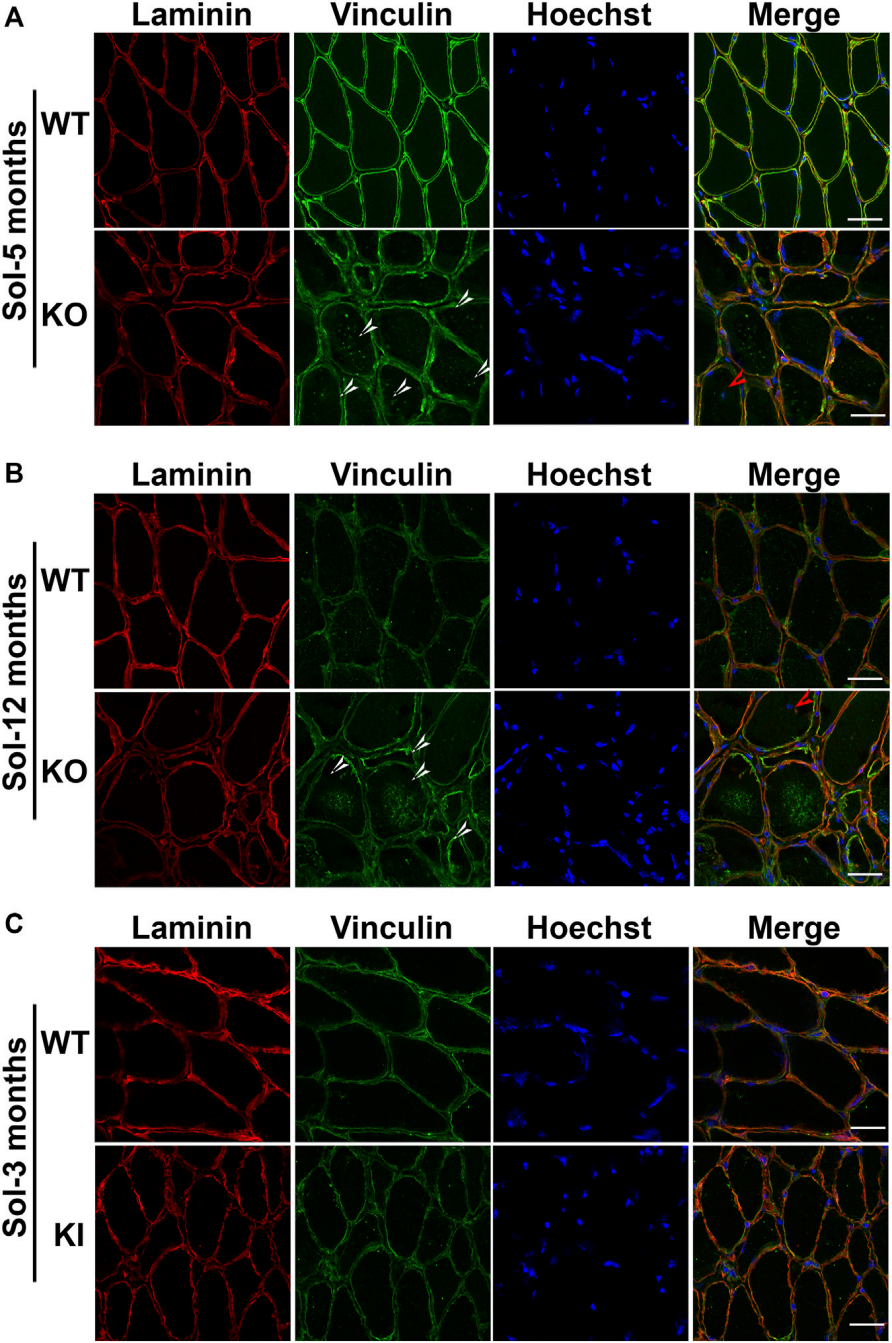
Localization of vinculin in the soleus of DesKO and DesKI-R405W mice. Microscopic images of soleus cross sections from DesKO mice at 5 months **(A)** and 1 year of age **(B)** and DesKI-R405W 3-month-old mice **(C)**. Vinculin accumulations in and around the fibers are shown by the white arrows and central nuclei by red arrows. Laminin was stained in red, vinculin in green and nuclei in blue. Images were taken with a ×40 objective, and the scale bar corresponds to 30 μm.

To determine if this higher expression level is associated with altered localization of vinculin in mature muscle, we performed immunostaining on soleus muscles. First, as expected, desmin labeling was detected in muscle sections from WT but not DesKO mice (Supplementary Figure S6A). Second, soleus from 5-month-old WT mice showed a well-localized laminin layer around the muscle fibers (Figure 8A). In addition, vinculin was observed at the membrane, with homogeneous staining all around the fiber periphery. In contrast, in the soleus of homozygous DesKO mice, the muscle fibers appeared disorganized, with varying size and an increased interfiber space, suggesting dystrophy and fibrosis as previously described (Li et al., 1997; Agbulut et al., 2001). Moreover, the vinculin immunostaining along the sarcolemma presented some heterogeneity in DesKO mice, with more intense areas at the membrane (white arrows, Figure 8A). In addition, some vinculin was surprisingly located inside the fibers (white arrows, Figure 8A), which was never observed in WT mouse muscles.

Fibers in the soleus of 1-year-old DesKO mice appeared more affected than those of 5-month-old DesKO mice. In addition to internalized nuclei (red arrows, Figure 8B), more fibers presented very variable size and shape, a sign of muscular dystrophy. Laminin labeling also showed an even greater increase in matrix space, possibly due to fibrosis. Interestingly, intrafibrillar accumulations of vinculin were more abundant (white arrows, Figure 8B). As in the 5-month-old muscles, vinculin staining was much more diffuse and heterogeneous along the membranes. The same characteristics were also observed in homozygous DesKO mouse diaphragms at 1 year of age (Supplementary Figure S6B).

In parallel, we analyzed vinculin localization in the soleus from 3-month-old DesKI-R405W mice (Figure 8C). Laminin staining revealed that fibers of homozygous DesKI-R405W mice were smaller than those of WT mice, suggesting muscle atrophy, as recently described (Herrmann et al., 2020). DesKO mouse muscle exhibit thickening of the matrix space between the fibers. Again, DesKI mice had more accumulations of the vinculin staining area (white arrows in Figure 8C) at the plasma membrane than WT mice. However, contrary to DesKO mice, no vinculin accumulation was detected inside the fibers of DesKI mice.

Together, these results suggest that vinculin increase is associated with mislocalization in adult muscle in both models.

### Vinculin Expression is Also Altered in Patients With Desminopathies Associated With a Total Lack of Desmin

**FIGURE 9:**
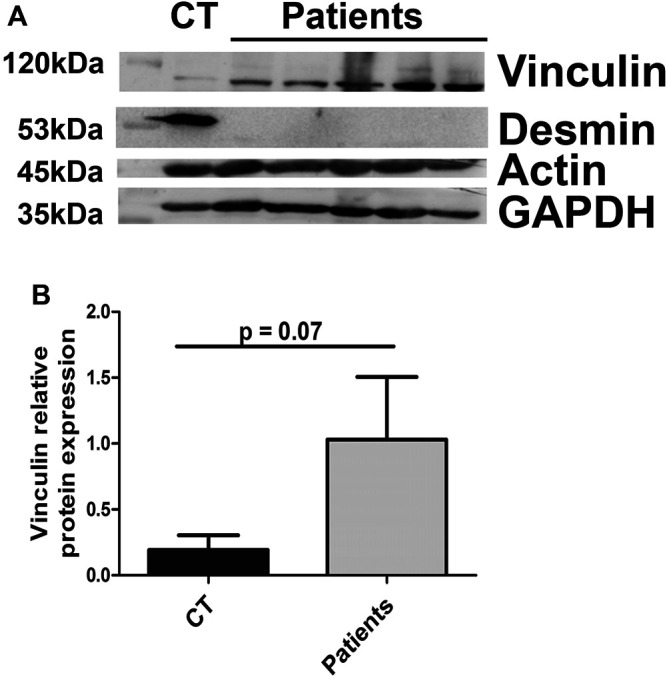
Vinculin relative protein expression in patient muscle biopsies. The amounts of vinculin, actin, and desmin were measured in muscle biopsies from controls (CT) or patients carrying homozygous mutations in the desmin gene. GAPDH was used as a loading control.

To see if vinculin expression modifications were also found in muscles of patients with pathological conditions, we obtained protein extracts from biopsies of patients carrying mutations leading to a total lack of desmin expression (Figure 9A). Indeed, the absence of desmin in patient tissue was associated with increased vinculin in the muscles (Figure 9). In contrast, the amount of actin was not disturbed (Figure 9).

## Discussion

### Desmin Regulates Various Signaling Pathways Including Adhesion

Desmin is well known as a specific IF essential for muscle integrity and function. However, desminopathies highlight other potential important roles of desmin. To investigate pathways associated with desmin expression, we performed a wide analysis of gene expression in stable C2C12 cells overexpressing WT or p.R406W desmin. As expected, we observed modifications in several genes encoding proteins which are able to co-polymerize with desmin (Hol and Capetanaki, 2017) and sarcomeres or are involved in contraction linked to specific desmin location at the Z-disk.

Surprisingly, desmin network perturbation also led to alterations of gene expression associated with adhesion signaling. Specific cell adhesion molecules were upregulated, potentially leading to perturbation of cell adhesion properties (anchoring, moving and interactions with extracellular matrix). All these functions are essential for muscle, particularly during regeneration or force generation. Further, these processes are altered in DesKO mice (Agbulut et al., 2001). Taken together, our results highlight for the first time a potential new role of desmin in cell adhesion.

### Overexpression of Desmin Modulates Focal Adhesion Area in Electroporated C2C12 Cells

As cell interactions with the ECM are essential for adhesion, we first focused our study on the link between desmin IFs and FAs, the main adhesive cell complexes, in adherent undifferentiated C2C12 myoblasts. Overexpression of wild-type desmin in C2C12 myoblasts increases the total area of adhesion patches containing vinculin without increasing their number. These results suggest a gain of function for myoblasts enriched in wild-type desmin. Moreover the increase is more pronounced for the adhesion patches located at the center of the cells, suggesting that desmin could play a role in the maintenance of the focal adhesions. Yet, electroporation of p.R406W desmin does not modify the total area of adhesion patches, indicating a loss of function of mutated desmin. Similar results have been found by our lab concerning the role of desmin in the rigidity of the cells, with a higher rigidity of C2C12 cells overexpressing WT desmin but not of cells overexpressing p.E413K mutated desmin (Charrier et al., 2018). Thus, desmin could play a role in the development and/or maintenance of FAs, with this function lost in the presence of the mutation. In endothelial cells, vimentin participates in the maturation of FAs to allow better adhesion of cells subjected to hemodynamic stress (Tsuruta and Jones, 2003). Desmin and vimentin share a large homology (80%) and both proteins are expressed in myoblasts. However, overexpression of WT or mutated desmin does not modify the vimentin network (Charrier et al., 2016), nor expression (Figures 4I,J).

Thus, desmin could have the same role in FA regulation in undifferentiated myoblasts as vimentin in other cells. However, desmin seems to play at least a role in FA regulation, since the number of patches per cell is not affected by desmin mutation. Interestingly, FA size closely correlates with the local forces that cells develop on their substrates (Balaban et al., 2001). The present study, along with our previous findings regarding p.E413K desmin, a mutation which is associated with impaired contractility (Charrier et al., 2016), suggest that desmin mutation could impair the forces that myoblasts develop against their substrates by interfering with modulation of FAs.

It would also be interesting to compare the composition of FAs for cells overexpressing WT desmin vs. p.R406W mutated desmin, in particular regarding integrins or proteins associated with FA maturation. However, myoblasts are specific muscle cells and unfortunately the proteins tested (such as beta1 and alpha7 integrins, or Zyxin) did not provide sufficiently specific immunostaining to perform TIRFM experiments.

The p.R406W mutation is localized at the end of the 2B domain in the TYRKLLEGEE consensus domain that could have a role in maintaining structure and interactions with important proteins to modulate FAs. In particular, desmin interacts and co-polymerizes with synemin, a type VI intermediate filament also expressed in muscle. Synemin is also linked to FAs in muscle (Sun et al., 2008a; Sun et al., 2008b). However, synemin is totally excluded from aggregates of p.E401K, p.R406W, and p.E413K mutants overexpressed in C2C12 cells (Chourbagi et al., 2011). Thus, the effect of the p.R406W mutation on FA maturation could be due to a lack of or decreased interaction with synemin. Moreover, synemin presents altered localization and expression in the soleus in DesKO (Rodríguez et al., 2018) and DesKI-R349P (Clemen et al., 2015) mice, reinforcing the potential link between desmin network perturbation and the observed vinculin defects.

Furthermore, desmin interacts with several proteins in costameres, such as plectin, or syncoilin. These proteins are themselves linked to the dystrophin-glycoprotein complex, which is a specific adhesion complex complementary to FAs observed in muscle (Ervasti, 2003). Immunostaining of soleus muscles from 1-year-old DesKO mice showed some fibers presenting dystrophin accumulations at the membrane. Thus, desmin could affect not only FA regulation but also the other adhesion complexes through its various protein interactions, leading to costamere weakness.

### DesKO and DesKI Satellite Cells Exhibit Decreased Adhesive Properties on Fibronectin Substrate Under Hydrodynamic Flow

As FAs are involved in the strength of adhesive bonds between cells and the ECM, we decided to measure the strength of cell adhesion under shear stress, elicited by a flow with a fixed and constant speed. We have previously seen that desmin p.R406W presents a loss of function compared to WT desmin. Our results show that satellite cells lacking desmin or expressing p.R405W desmin detach more rapidly than WT cells. This suggests that these cells adhere less to the substrate, i.e., fibronectin, compared to control cells. These results are consistent with the TIRFM data obtained on C2C12 myoblasts, suggesting a loss of function of the R406W mutated desmin with respect to AF regulation.^1^ Alteration of FA maturation could lead to a decrease in their adhesive strength which would be characterized by lower resistance during shear-stress, increasing the rate of detachment. Moreover, DesKO satellite cells demonstrate that desmin absence leads to the same phenotype as the p.R405W mutation in satellite cells. This confirms the loss of function observed in the mutant compared to WT. In adhesion patches, integrins are considered essential contributors to the interaction with the substrate. Further, α5 integrin is crucial in muscle functions. Mice lacking this integrin chain have muscular dystrophies (Taverna et al., 1998). Moreover, keratin type I/II IFs can regulate integrin turnover (Seltmann et al., 2015). Thus, it is possible that desmin modifies FA adhesion, especially integrin clustering, directly or via its interaction with other IFs such as synemin or keratin.

### DesKO and DesKI-R405W Satellite Cells Exhibit Faster Migration

FAs assemble and disassemble in a dynamic fashion when cells are moving (Webb et al., 2002). We thus analyzed the migration of WT satellite cells or homozygous DesKO and DesKI-R405W cells. We chose to use fibronectin and laminin, the two main components of the extracellular matrix of skeletal muscle (Gillies and Lieber, 2011), which are essential during the muscle regeneration process (Repesh et al., 1982; Gulati et al., 1983; Lukjanenko et al., 2016), when satellite cells are activated. In particular, satellite cells synthetize fibronectin during their activation (Bentzinger et al., 2012), reinforcing the hypothesis that fibronectin is the primordial substrate for their adhesion and laminin is more important as a substrate for migration. As expected, satellite cells migrated faster on laminin than on fibronectin regardless of the genotype of the cells (WT, DesKO or DesKI-R405W) (Siegel et al., 2009). Homozygous DesKI-R405W and DesKO satellite cells migrated faster than WT cells on both fibronectin and laminin.

As migration is in part linked to the adhesion process, the difference in migration speed observed in mutant cells (DesKO and DesKI-R405W) could be due to decreased interaction with the matrix. Indeed, changes in the cell-laminin and cell-fibronectin interactions of the DesKO and DesKI-R405W satellite cells would explain their faster movement. This hypothesis aligns with our previous observations showing alterations in vinculin patch size in cells overexpressing desmin p.R406W and decreased adhesion of DesKI-R405W and DesKO satellite cells to fibronectin. However contratry to FA regulation, desmin seems to have opposite effect on migration than vimentin. Indeed, vimentin KO cells exhibits slower migration rate (Eckes et al., 2000) and overexpression of vimentin lead to increase migration speed (Mendez et al., 2010). Moreover vimentin is considered as an epithelio-mesenchymal transition (EMT) marker and facilitates invasion of mesenchymal cells by controlling the dynamics and distribution of FAs (Mendez et al., 2010; Menko et al., 2014; De Pascalis et al., 2018). In contrast keratin 6 KO cells lead to a decrease of migration rate by modulating FA pathways (Wong and Coulombe, 2003; Rotty and Coulombe, 2012; Wang et al., 2018). Desmin (type III IF) is closer to vimentin (type III IF) than to keratins (type I and II IF), suggesting at first sight a more similar role. However, in muscle, desmin is upregulated during differentiation and vimentin decreased suggesting specific role of desmin in this tissue. Moreover, we did not see modification of vimentin expression level in desKO or desKI satellite cells (Figures 3I,J), that could suggest a compensation leading to the increase of migration rate. Thus regarding FA modulation or migration, vimentin and desmin could have some distinct roles by interacting with specific partners. Finally in the microarray, vimentin or synemin gene does not appear to be regulated, whereas keratin one seems to be modulated (Supplementary Table S2). Depending on the cell type, IFs have different effects on cell migration that may be explained by the difference in IF proteins or integrin expression patterns. Keratin 18 and 19 are known to be expressed in mature muscle and could be associated with desmin (Muriel et al., 2020). Moreover Keratin 16 overexpression has already been demonstrated linked to increase cell migration in cancer (Yuanhua et al., 2019)**.** Altogether, keratins in association with desmin would be a potential pathway to explore. However we did not succeed in identifying keratins expression in undifferentiated myoblasts and satellite cells, maybe their expression level is too low to be detected or antibody conditions need to be adapted. Indeed these keratins have been described in mature muscle with an mRNA level at least 1,000 times lower than that of desmin (Muriel et al., 2020). Desmin expression is low in satelitte cells or undifferentiated myoblasts and increases during differentiation and it could be the same for keratins. Thus some optimization is needed to confirm the potential role of keratins in future work. Altogether, keratins in association with desmin would be a potential pathway to explore in future work.

We also studied the persistence of the satellite cells. No difference was observed between the cell genotypes on laminin. However, on fibronectin, the DesKO satellite cells presented decreased persistence compared to WT and DesKI-R405W cells. Interestingly, vimentin knock-out also leads to downregulation of persistence during wound healing experiments (Eckes et al., 2000). Thus, desmin seems to have a similar function in myoblasts to that of vimentin in other cell types. It is therefore consistent that desmin invalidation could affect cell persistence. Moreover, vimentin expression is not increased upon desmin KO (Capetanaki et al., 1997; Li et al., 1997). Thus, vimentin cannot compensate for the loss of skeletal-muscle-specific desmin.

The difference between the two types of mutant cells (DesKO and DesKI-R405W) could be due their differential effects on the interactions of desmin and its partner. The mutation localized at the end of the 2B domain could potentially alter some interactions (as with synemin) but not others, whereas the absence of desmin modifies its entire interactome. In fact, phenotypes of DesKI-R405W mice are not totally similar those of DesKO mice, even if they share some similarities, suggesting a mutant-specific effect.

It is difficult to extrapolate the adhesive properties and migration of satellite cells to mature muscle. Indeed, *in vivo*, faster migration speed would allow satellite cells to reach the injury site to regenerate the tissue more rapidly. However, if satellite cells continue migrating for too long, this could impair their ability to fuse with the fibers to restore the muscle, especially with the decrease of cell-matrix adhesion. Indeed, defects in regeneration, particularly a delay in fiber maturation, are described in DesKO mice (Agbulut et al., 2001). This would be consistent with defective satellite cell adhesion. Moreover, in the case of DesKO, the directionality seems to also be affected. This could therefore lead to an error or delay in addressing satellite cells to the location of the injury that could partly explain the observed regeneration defects in DesKO mice.

### Vinculin Expression and Localization Are Altered in DesKO and DesKI-R405W Muscle

We further quantified the expression of vinculin in mature skeletal muscle. In our model, desmin expression is increased by around two fold in the quadriceps and soleus muscle of 3-month-old homozygous DesKI-R405W mice compared to WT mice (Herrmann et al., 2020). Vinculin expression increased in an age-dependent manner in DesKO mice (quadriceps and soleus). This increase also occurred in the soleus of 3-month-old DesKI-R405W mice. At first, this alteration seems to contradict previous results obtained *in vitro* showing a decrease in the area of cellular FAs associated with weaker adhesion of satellite cells, which also does not present modifications of vinculin expression. However, the amount of vinculin increases during aging, especially in cardiac cells (DeLeon-Pennell and Lindsey, 2015). In addition, vinculin can regulate cell stiffness due to its ability to form adhesion patches (Auernheimer and Goldmann, 2014). Thus, overexpression of vinculin could lead to increased muscle rigidity. This increase could also be a compensation effect against, in part, costamere fragility which could rise over time due to the progressive degradation of muscle fibers. Indeed, at least in the DesKO model, focal disorganizations inside the muscle have been described and could induce costamere fragility (Li et al., 1996). In response to this alteration, muscle fibers would overexpress vinculin and maybe some other costamere proteins to try to maintain adhesion.

In parallel, we determined the localization of vinculin in DesKO or DesKI-R405W fibers. In normal muscle, vinculin is located at the costamere, at the interface between the membrane and endomysium; yet, vinculin shows an increased expression level as well as mislocalization with peripheral accumulation for both models and intramyofibrillar accumulation in DesKO muscles. These vinculin intrafibrillar deposits seem to worsen over time according to our observations in 1-year-old soleus. Vinculin accumulations were found only at the periphery and not inside the fibers in DesKI-R405W mouse muscles. This may be partly explained by the fact that mice die at the age of 3 months. Indeed, the muscular injury may not have been fully developed yet. However, as the accumulations are inside DesKO fibers, they therefore are totally independent of desmin aggregation (as found in DesKI-R405W). Moreover, we know that the desmin-rich aggregates found in patients with desminopathies are composed of several proteins captured during their formation. Laser microdissection of aggregates followed by proteomic analysis performed in five patients with different mutations in the desmin gene did not demonstrate the presence of vinculin in these formations (Maerkens et al., 2013). Finally, preliminary results from muscle biopsies of patients with mutations leading to desmin invalidation also show an increase in vinculin. These results suggest that the disturbed localization of vinculin is not due to the presence of aggregates in the fibers but rather to the disorganization of the desmin network itself.

In sum, these results suggest that disruption in the desmin network could lead to alteration of muscle fiber adhesion. Physiologically, the decrease in adhesion between fibers and extracellular matrix as well as vinculin mislocalization and the defects of satellite cell adhesion and migration could lead to reduced force transmission produced by muscles. Indeed, disorganization of sarcomeres due to the lack or presence of desmin aggregates generates a defect in force production (Li et al., 1997; Anderson et al., 2002) that causes the muscle weakness observed in patients. However, force transmission is not only longitudinal to the tendon (Sharafi and Blemker, 2011), but also lateral from costameres to the endomysium (Zhang and Gao, 2014). Thus, reduced force production and altered force transmission would generate significant muscle weakness in patients. In addition, the adhesion and migration defects observed in satellite cells could lead to decreased muscle regeneration and efficiency.

## Conclusion

**FIGURE 10:**
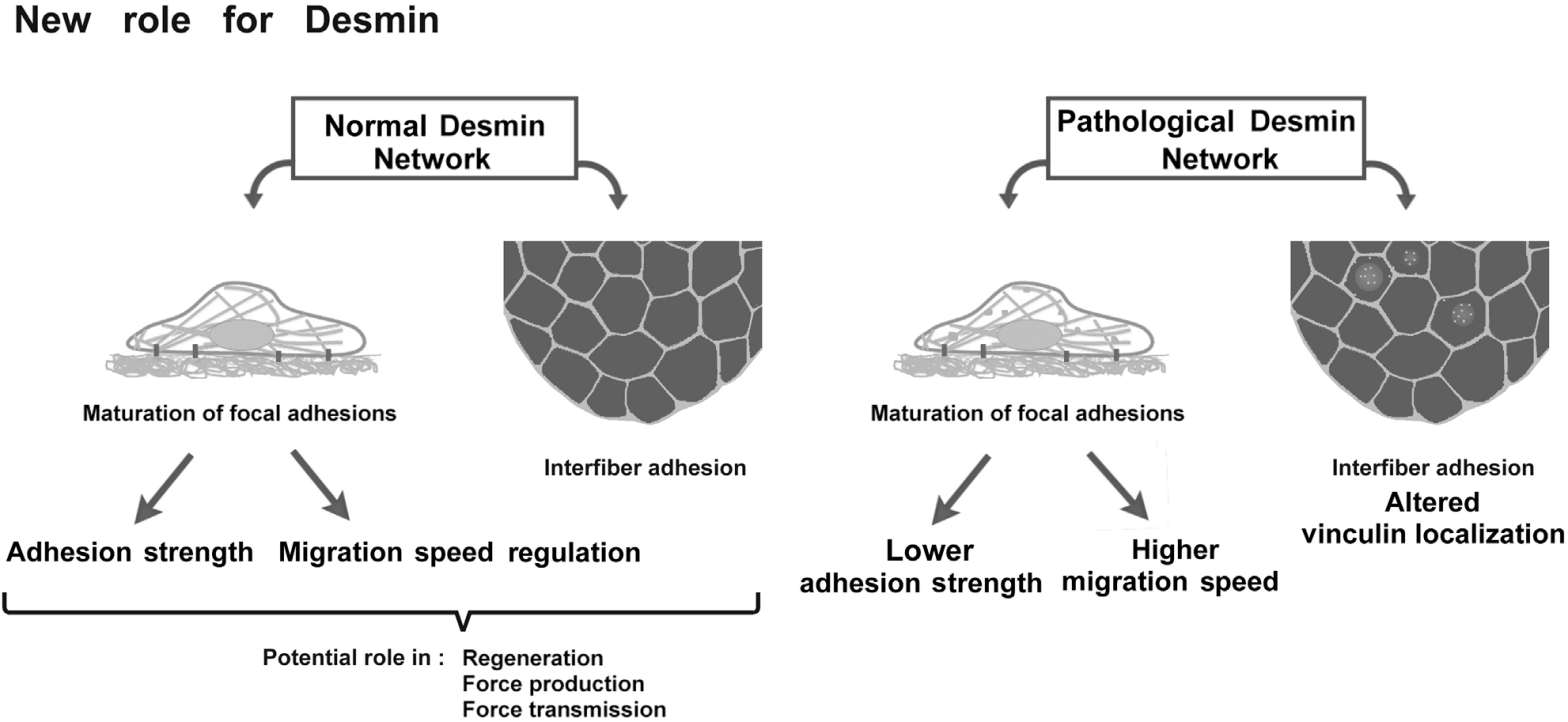
Schematic representation of desmin roles in adhesion and migration and the potential consequences on muscle.

This study demonstrates that disruption of desmin leads to defects in skeletal muscle cell adhesion and migration (Figure 10). This new role of desmin opens avenues of investigation into the physiology and pathology of desminopathies, potentially leading to the identification of new therapeutic targets for these currently incurable diseases.

## Data Availability

The datasets presented in this study can be found in online repositories. The names of the repository/repositories and accession number(s) can be found below: Gene Expression Omnibus, GSE185589.

## Footnotes

1 Note that TIRFM experiments could unfortunately not be reproduced on satellite cells, due to their poor attachment to the glass coverslips, a necessary experimental condition for TIRFM.

## Supplementary Material

Supplementary Figure 1 Method for measuring focal adhesion area. **(A)** Observation of vinculin by TIRFM allows visualization of adhesion patches. Epifluorescence and TIRFM images were taken with the ×40 objective. Scale bar = 10 µm. **(B)** ImageJ software was used to measure the area of adhesion. The four steps used for image processing are listed.

Supplementary Figure 2 Relative amount of transfected desmin compared to endogenous in electroporated C2C12 cells. The amount of GFP-WT and GFP-R406W desmin was measured in C2C12 cells, 24 h after electroporation. Endogenous and exogenous (GFP-constructs) was detected by anti-desmin antibody. Each histogram represents the average relative protein expression of four independent experiments normalized by electroporation rate. Measured on >100 cells. The error bars represent the SD.

Supplementary Figure 3 Mean migration rates of satellite cells according to the substrate. Mean migration rates of WT **(A)**, DesKO **(B)** and DesKI-R405W **(C)** cells are plotted against the substrate on which they are adhered. Each point represents the average speed of a cell and the error bars represent the standard deviation. A non-parametric Mann-Whitney test was performed: ****p* < 0.001.

Supplementary Figure 4 Relative vinculin protein expression in DesKO mouse quadriceps. The amounts of vinculin **(B,E,H)** and metavinculin **(C,F,I)** were measured in quadriceps of mutant DesKO mice at 3 months **(A–C)**, 5 months **(D–F)** and 1 year **(G–I)** of age. Each histogram represents the average relative protein expression of four independent GAPDH-standardized experiments for more than 5 WT mice and five DesKO mice (3 months: 8 WT and 6 DesKO; 5 months: 6 WT and 6 DesKO; 1 year: 6 WT and 5 DesKO). A non-parametric Mann-Whitney test was performed on the samples. All conditions are not significant with **p* < 0.05; ***p* < 0.01; ****p* < 0.001. The error bars represent the SD.

Supplementary Figure 5 Relative vinculin and desmin protein expression in 3-month-old DesKI-R405W mouse quadriceps. The amount of desmin **(A,B)** and vinculin **(C,D)** was measured in quadriceps of DesKI-R405W mice at 3 months. Each histogram represents the average relative protein expression of three independent GAPDH-standardized experiments from 5 WT and 2 DesKI-R405W mice. Vinculin (120 kDa) and desmin (53 KDa) are revealed on the same gel than GAPDH (35 KDa), which are consequently presented twice on the panel **(A, C)**. A non-parametric Mann-Whitney test was performed on the samples with ***p* < 0.01; ****p* < 0.001. The error bars represent the SD.

Supplementary Figure 6 Protein localization in muscles of 1-year-old DesKO mice. Microscopic images of cross sections of the soleus **(A)** and diaphragm **(B)**. **(A)** Desmin (green) is found on the edges of the fibers as well as inside the fibers of WT mice (white arrows). No desmin labeling is found in homozygous DesKO individuals. Laminin is stained in red, nuclei are in blue. **(B)** Vinculin (green) accumulations and a thickening of vinculin labeling at the fiber membrane are shown by white arrows. Laminin is marked in red, and nuclei in blue. The confocal images were taken with a ×40 objective and the scale bar corresponds to 30 μm.

## Abbreviations

BSA, bovine serum albumin; Cy3-, 5-, Cyanine Dyes 3-, 5-; DMEM, Dulbecco’s Modified Eagle’s Medium; ECL, enhanced chemiluminescence; EGFP, enhanced green fluorescent protein; FA, focal adhesions; FBS, fetal bovine serum; GAPDH, glyceraldhyde-3-phosphate dehydrogenase; IF, intermediate filament; PBS, phosphate buffered saline; SDS–PAGE, sodium dodecyl sulfate–polyacrylamide gel electrophoresis; TIRFM, total internal reflection fluorescence microscope.
