# Supplementary figures and images for: Desmin Modulates Muscle Cell Adhesion and Migration
Source: Front Cell Dev Biol. 2022 Mar 8;10:783724. doi: 10.3389/fcell.2022.783724 (PMC8957967; doi:10.3389/fcell.2022.783724)

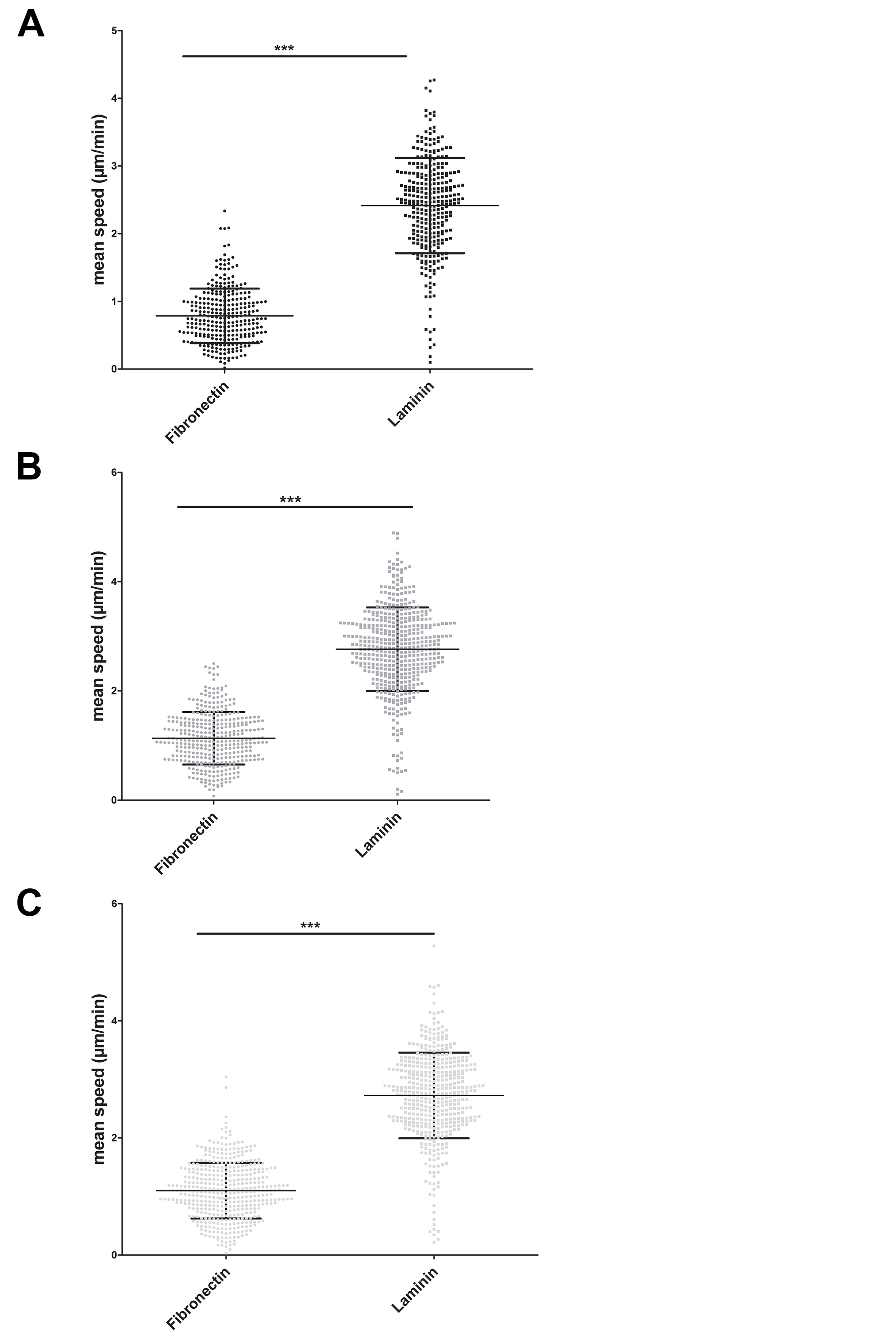

Supplement: Supplementary file 2 [file Image3.JPEG]

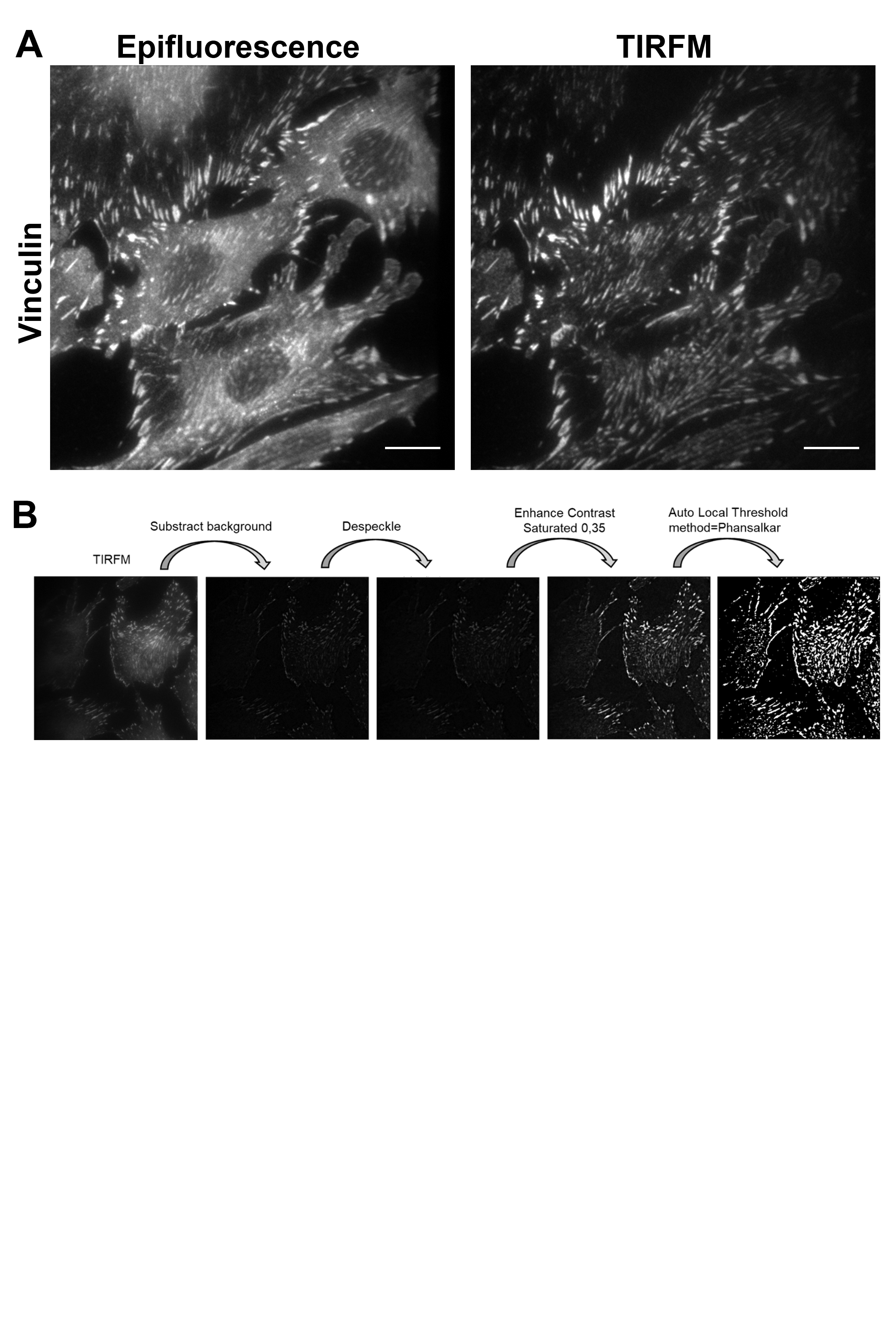

Supplement: Supplementary file 5 [file Image1.JPEG]

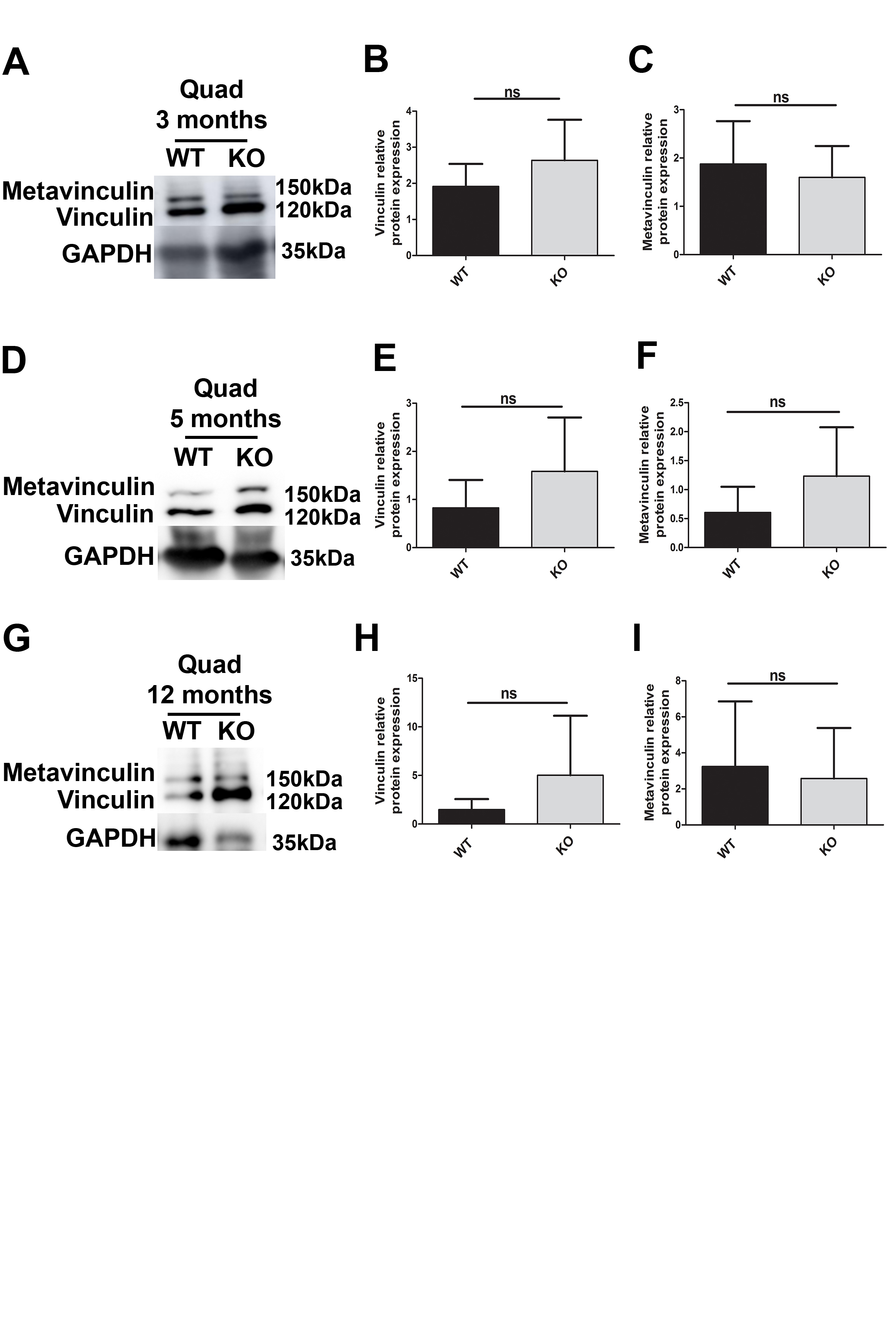

Supplement: Supplementary file 6 [file Image4.JPEG]

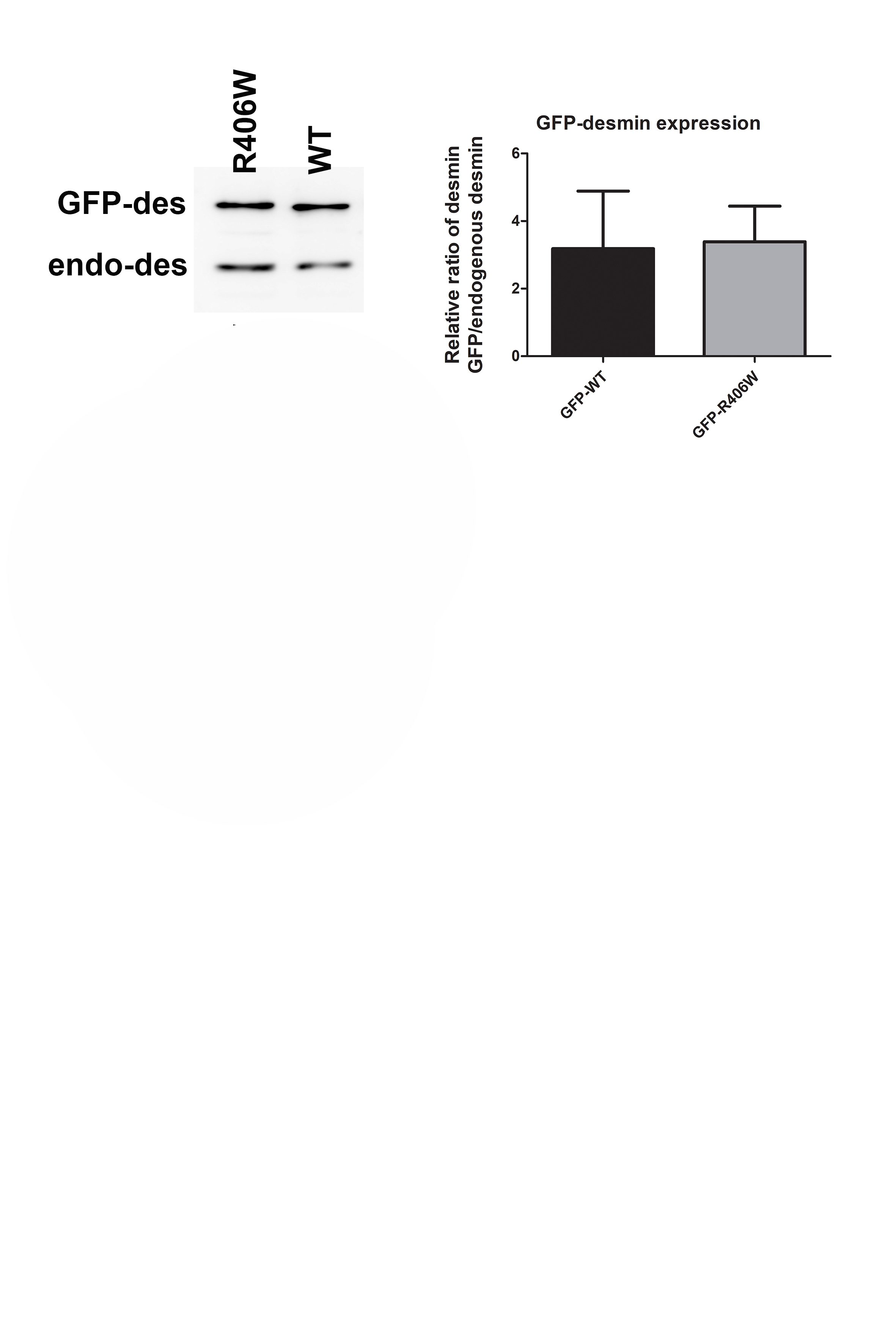

Supplement: Supplementary file 7 [file Image2.JPEG]

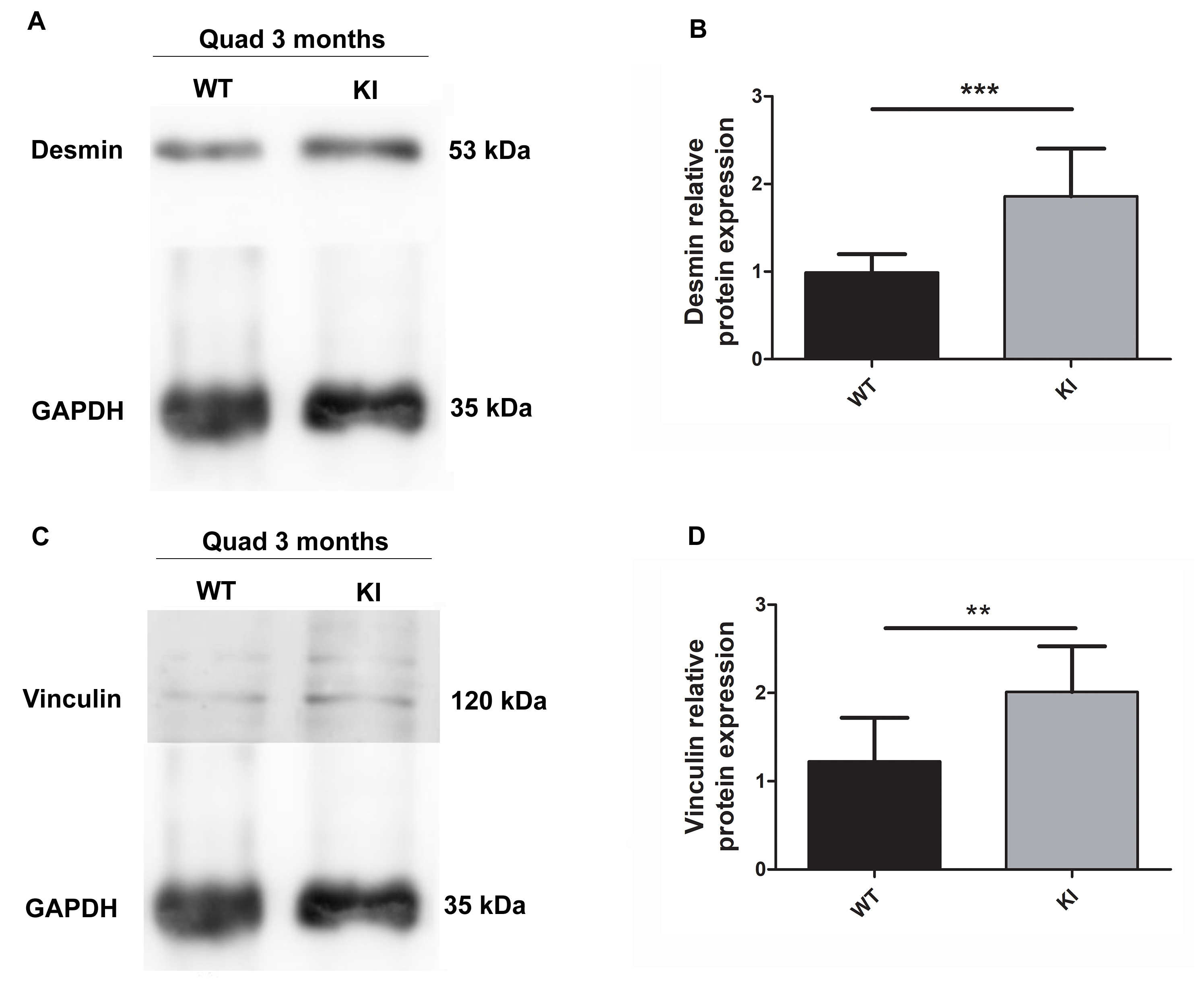

Supplement: Supplementary file 8 [file Image5.JPEG]

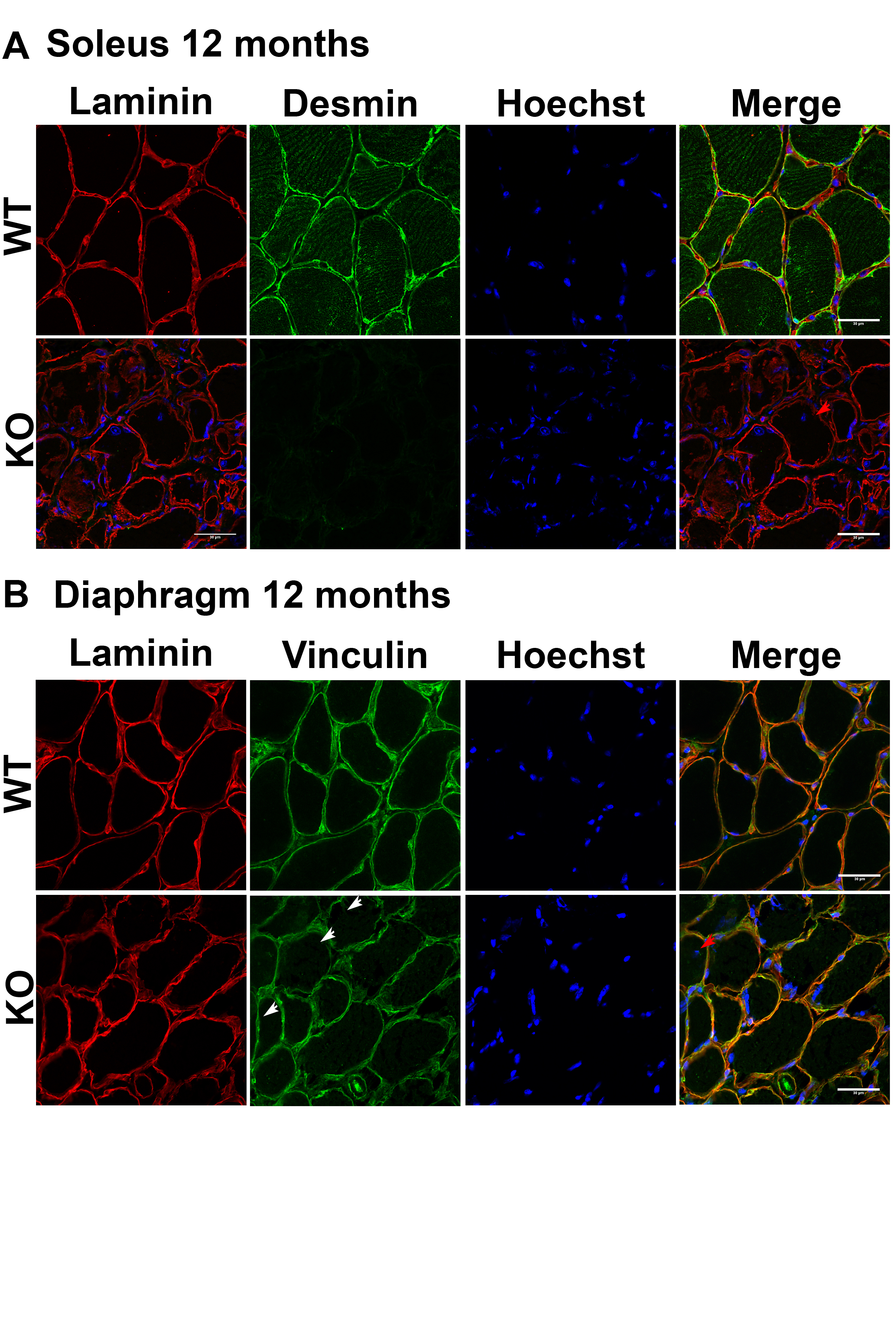

Supplement: Supplementary file 11 [file Image6.JPEG]
