# Supplementary material for: Desmin Modulates Muscle Cell Adhesion and Migration
Source: Front Cell Dev Biol. 2022 Mar 8;10:783724. doi: 10.3389/fcell.2022.783724 (PMC8957967; doi:10.3389/fcell.2022.783724)
Supplement: Supplementary file 4 [file Table1.DOCX]

A.

| **Upregulated genes of cell adhesion cluster** | **ID (Entrez database)** | **Fold Change** | **p-value** |
| --- | --- | --- | --- |
| catenin (cadherin associated protein), alpha-like 1 | 54366 | 2.00997 | 8.53554E-6 |
| mannan-binding lectin serine peptidase 2 | 17175 | 2.0755 | 0.00544 |
| protocadherin 9 | 211712 | 15.04775 | 7.05313E-12 |
| selectin, platelet | 20344 | 2.79664 | 9.90042E-20 |
| cadherin 10 | 320873 | 2.08627 | 0.00104 |
| riken cdna 1500004i01 gene | 76884 | 3.22349 | 4.01175E-6 |
| sry-box containing gene 9 | 20682 | 2.17757 | 0.00003 |
| plakophilin 3 | 56460 | 2.80314 | 1.35017E-7 |
| adhesion molecule with ig like domain 2 | 105827 | 3.29514 | 1.06977E-9 |
| neuronal growth regulator 1 | 320840 | 5.04804 | 3.33141E-7 |
| collectin sub-family member 10 | 239447 | 4.54577 | 1.74293E-12 |
| interleukin 18 | 16173 | 2.02574 | 4.38582E-10 |
| cadherin 13 | 12554 | 2.28908 | 2.46434E-7 |
| laminin, gamma 2 | 16782 | 2.04213 | 4.92679E-6 |
| fibronectin leucine rich transmembrane protein 3 | 71436 | 6.95414 | 0.00018 |
| stabilin 1 | 192187 | 3.46421 | 0.00009 |
| mesothelin | 56047 | 3.74525 | 1.42329E-12 |
| transcription factor ap-2, alpha | 21418 | 5.80643 | 5.33348E-16 |
| integrin beta 7 | 16421 | 2.03624 | 4.93587E-6 |
| hypothetical protein 9430004m15 | 329628 | 2.37253 | 1.01265E-7 |
| testicular cell adhesion molecule 1 | 75870 | 2.62447 | 3.09355E-8 |
| otoraplin | 57329 | 3.2898 | 3.55913E-11 |

B.

| **Downregulated genes of cell adhesion cluster** | **ID (Entrez database)** | **Fold Change** | **p-value** |
| --- | --- | --- | --- |
| cadherin 15 | 12555 | -5.36823 | 1.25178E-15 |
| transglutaminase 2, c polypeptide | 21817 | -4.28279 | 3.92231E-11 |
| protocadherin beta 20 | 93891 | -2.29136 | 2.63510E-7 |
| protocadherin beta 21 | 93892 | -2.01354 | 6.59937E-6 |
| perp, tp53 apoptosis effector | 64058 | -3.5212 | 3.03033E-6 |
| laminin, beta 3 | 16780 | -3.54936 | 5.96143E-11 |
| protocadherin beta 15 | 93886 | -2.14415 | 0.00010 |
| jagged 2 | 16450 | -2.87014 | 2.46978E-9 |
| procollagen, type i, alpha 1 | 12842 | -7.29315 | 0 |
| protocadherin beta 22 | 93893 | -2.03558 | 5.17216E-6 |
| procollagen, type v, alpha 3 | 53867 | -2.74833 | 2.26938E-9 |
| elastin microfibril interfacer 2 | 246707 | -2.20412 | 6.52665E-7 |
| transforming growth factor, beta induced | 21810 | -25.75762 | 9.17352E-22 |
| sialophorin | 20737 | -2.02996 | 5.64481E-6 |
| procollagen, type vi, alpha 3 | 12835 | -2.14277 | 1.34649E-6 |

| procollagen, type xiv, alpha 1 | 12818 | -4.76634 | 1.41561E-11 |
| --- | --- | --- | --- |
| tumor necrosis factor alpha induced protein 6 | 21930 | -2.83077 | 2.36505E-9 |
| cadherin 11 | 12552 | -4.66491 | 1.56944E-14 |
| laminin, alpha 1 | 16772 | -7.7375 | 1.56469E-8 |
| angiotensinogen (serpin peptidase inhibitor, clade a,  member 8) | 11606 | -9.56217 | 4.41598E-15 |
| sorbin and sh3 domain containing 1 | 20411 | -2.0784 | 2.93228E-6 |
| procollagen, type iii, alpha 1 | 12825 | -2.80009 | 1.41134E-9 |
| epidermal growth factor receptor | 13649 | -2.04363 | 2.66064E-10 |
| protocadherin beta 16 | 93887 | -2.10006 | 3.99333E-6 |
| integrin, alpha 11 | 319480 | -3.72127 | 1.64362E-12 |
| periostin, osteoblast specific factor | 50706 | -4.77335 | 1.02457E-14 |
| laminin, alpha 2 | 16773 | -2.41689 | 1.16807E-7 |
| protein tyrosine phosphatase, non-receptor type  substrate 1 | 19261 | -4.43306 | 4.59539E-14 |
| tenascin c | 21923 | -5.55774 | 1.98585E-11 |
| cd34 antigen | 12490 | -3.43861 | 9.81711E-12 |
| apoptotic peptidase activating factor 1 | 11783 | -2.97299 | 0.00945 |
| procollagen, type viii, alpha 1 | 12837 | -2.47659 | 3.22957E-8 |
| fibulin 5 | 23876 | -2.24433 | 4.10235E-7 |
| myosin binding protein h | 53311 | -8.55058 | 4.79177E-14 |
| laminin, alpha 4 | 16775 | -2.38023 | 1.37258E-7 |
| procollagen, type xxiv, alpha 1 | 71355 | -2.88839 | 3.28057E-9 |
| procollagen, type ii, alpha 1 | 12824 | -2.63787 | 1.00466E-17 |

**Supplemental table 1. Up-regulated and down-regulated genes from the Cell Adhesion cluster.**

Up-regulated and down-regulated genes of C2C12 expressing R406W desmin were sorted compared to C2C12 expressing WT desmin. Then, they were clustered using gene ontology enrichment analysis and genes up-regulated (A) or down-regulated (B) of the Cell Adhesion cluster are presented.
