# Supplementary material for: Desmin Modulates Muscle Cell Adhesion and Migration
Source: Front Cell Dev Biol. 2022 Mar 8;10:783724. doi: 10.3389/fcell.2022.783724 (PMC8957967; doi:10.3389/fcell.2022.783724)
Supplement: Supplementary file 9 [file Table2.DOCX]

| **Up-regulated genes of cell adhesion cluster** | **ID (Entrez**  **database)** | **Fold**  **Change** | **p-value** |
| --- | --- | --- | --- |
| keratin complex 1, acidic, gene 13 | 16663 | +2.11883 | 3.98578E-6 |
| keratin complex 1, acidic, gene 16 | 16666 | +2.39044 | 9.20368E-8 |
| keratin complex 1, acidic, gene 19 | 16669 | +2.43233 | 7.88605E-8 |
| beaded filament structural protein 2, phakinin | 107993 | +3.07882 | 0.00017 |
| keratin complex 1, acidic, gene 15 | 16665 | +6.60682 | 1.21617E-16 |
